# SeqUIaSCOPE: multi-omics data integration platform for single-patient clinical oncology pathway exploration

**DOI:** 10.1093/bioinformatics/btag630

**Published:** 2026-08-20

**Authors:** Kateřina Jurásková, Petra Pokorná, Kristián Kováč, Nicolas Blavet, Anna Molíková, Martin Demko, Vojtěch Bystrý

**Affiliations:** Central European Institute of Technology, Masaryk University, Brno 625 00, Czech Republic; National Centre for Biomolecular Research, Faculty of Science, Masaryk University, Brno 625 00, Czech Republic; Central European Institute of Technology, Masaryk University, Brno 625 00, Czech Republic; Department of Biology, Faculty of Medicine, Masaryk University, Brno 625 00, Czech Republic; Institute of Computer Science, Masaryk University, Brno 628 00, Czech Republic; Central European Institute of Technology, Masaryk University, Brno 625 00, Czech Republic; Central European Institute of Technology, Masaryk University, Brno 625 00, Czech Republic; Department of Biology, Faculty of Medicine, Masaryk University, Brno 625 00, Czech Republic; Central European Institute of Technology, Masaryk University, Brno 625 00, Czech Republic; Central European Institute of Technology, Masaryk University, Brno 625 00, Czech Republic

## Abstract

**Summary:**

SeqUIaSCOPE is an open-source platform designed for routine clinical oncology diagnostics through case-centric integration and visualization of genomic variants, fusion events, and expression profiles. The platform combines molecular-level validation via embedded genome browsing with systems-level interpretation through dynamic pathway visualization, enabling geneticists to assess how alterations converge across biological networks. Flexible reporting with customizable templates accommodates diverse institutional requirements, while secure cluster-based or local deployment ensures compliance with data protection policies, making advanced multi-omics diagnostics accessible to academic and clinical institutions.

**Availability and Implementation:**

SeqUIaSCOPE is freely available on GitHub at https://github.com/BioIT-CEITEC/sequiascope under the MIT license and archived at Zenodo (https://zenodo.org/records/21338445). Due to the sensitive nature of patient data, the repository provides simulated datasets that mimic the structure of real clinical data for testing and exploration. Documentation and a live demo accompany these datasets, allowing users to explore the application without any prior setup. The repository also includes a Helm chart for Kubernetes deployment and Docker containers for local deployment, ensuring compatibility across Linux, macOS, and Windows. No user registration is required, and all data remains on local or institutional infrastructure.

## 1 Introduction

Precision oncology diagnostics routinely produce large amounts of heterogeneous molecular data. To comprehensively capture tumor biological complexity, modern oncology practice has shifted from single-platform genomics toward integrated multiomics approaches. This expansion enables clinical decisions to be informed by diverse layers of biological information, creating new possibilities for personalized cancer care. However, the integration and interpretation of these complex multiomics datasets for individual patient diagnosis remains a significant challenge. Clinical geneticists currently face the demanding task of manually integrating information from multiple sources, such as genomic variants, expression profiles, pathway alterations, and relevant literature, into coherent diagnostic reports. This labor-intensive process is not only time-consuming but also prone to inconsistencies, particularly as the volume and complexity of molecular data continue to grow. There is a clear need for dedicated tools that can facilitate the comprehensive analysis of multiomics data in a case-centric framework.

Existing tools fall into three categories. First, specialized clinical software such as VarSome Clinical (Kopanos *et al.* 2019) focus on single-data type interpretation and lack the capability to accept or integrate multiomics datasets. This fragmentation forces clinicians to manually synthesize information across disparate platforms, increasing cognitive load and the risk of overlooking important molecular interactions across omics layers that may be critical for clinical decision-making. Second, cohort-based research platforms such as cBioPortal (Cerami *et al.* 2012) and UCSC Xena (Goldman *et al.* 2020) are designed for population-level research. While these multi-omics platforms excel at comparative analyses across large patient cohorts and are invaluable for discovery science, they are not designed for the detailed, iterative interpretation of individual patient cases required in clinical diagnostics. Third, commercial platforms such as SOPHiA DDM (Sophia Genetics n.d.) offer end-to-end integration and automated clinical reporting. However, these proprietary systems may present challenges regarding the transparency and limited customization, along with substantial licensing costs, making them less accessible for academic centers and collaborative research. Additionally, although some platforms offer local installation, many platforms remain exclusively web-based, requiring users to upload sensitive clinical data to external servers. That may be incompatible with institutional data protection policies and regulatory requirements. However, an open-source platform that supports routine clinical diagnostics while enabling comprehensive integration and interpretation of diverse molecular data layers for individual patient evaluation, without compromising data security, is currently unavailable.

**Figure 1:**
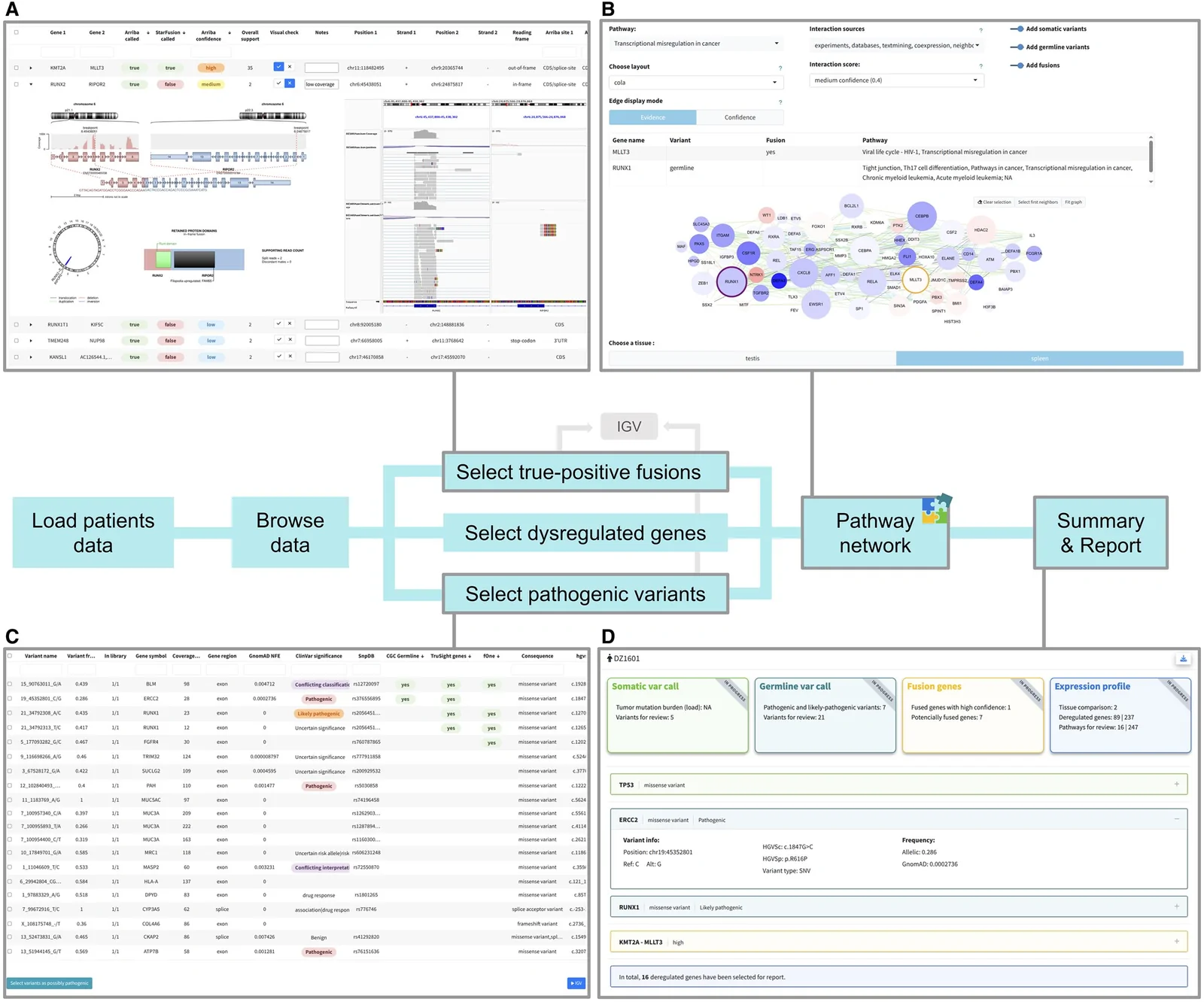
Schematic illustration of the SeqUIaSCOPE interface and intended workflow from data upload through variant/fusion/expression data exploration (with IGV validation) to pathway integration and final reporting. (A) Fusion gene module. (B) Pathway network module. (C) Germline variant calling module. (D) Summary and report module. Detailed descriptions of each module are provided in the documentation available in the GitHub repository and included as Supplementary Material.

We present SeqUIaSCOPE (Fig. 1), an open-source web-based platform designed for case-centric integration, visualization, and interpretation of multi-omics data in diagnostic oncology. SeqUIaSCOPE allows the evaluation of combined results from somatic and germline variant calling, fusion gene detection, and gene expression profiling for comprehensive single-patient assessment. A key strength of SeqUIaSCOPE lies in its integration of complementary data visualization approaches that enable both molecular-level validation and systems-level interpretation. The platform incorporates embedded genome browsing for real-time sequence-level validation of detected variants. This enables geneticists to visually inspect and confirm reported alterations directly within the platform, which is essential for any clinical application. Additionally, SeqUIaSCOPE also provides dynamic pathway visualization that contextualizes molecular alterations within functional biological networks. This network-level view enables clinical geneticists to identify systematic effects across multiple molecular layers, assess how gene variants and transcriptomic changes converge on shared pathways, and evaluate the functional consequences of genomic alterations within their broader regulatory context. Combining transcriptomic impact with variant and fusion findings in a single interaction network addresses a dimension typically absent from clinical workflows focused primarily on variant interpretation, giving geneticists a systems-level context in which to interpret individual alterations.

Built on an R Shiny framework with containerization, SeqUIaSCOPE enables scalable cluster-based deployment via Kubernetes Helm charts and local deployment via Docker, maintaining data security and compliance with institutional data protection policies and diagnostic standards. By combining research-grade multi-omics integration capabilities with practical features essential for clinical workflows, SeqUIaSCOPE bridges the gap between cohort-based research platforms and the operational needs of routine diagnostic practice.

## 2. Methods

### 2.1 Upload data

SeqUIaSCOPE enables the upload of multiple data types for individual patients, including somatic and germline variant calling results, fusion detection outputs, and gene expression profiles. The platform automatically validates file presence and required data columns for user-selected datasets, ensuring data completeness before processing.

### 2.2 Individual omics evaluation

Each data type is processed through dedicated interactive modules that present results in filterable tables and enable flagging of clinically relevant molecular alterations for detailed examination and inclusion in the final clinical report. Each dataset review can be marked complete, with progress tracked across modules in the interface. The case-centric design supports single-patient diagnosis while allowing multi-patient data management. The platform integrates built-in IGV (Robinson *et al.* 2023) to enable real-time validation of flagged variants and fusion breakpoints, providing direct inspection of sequence-level evidence. To ensure continuity in patient case analysis, SeqUIaSCOPE supports session saving and reloading, enabling seamless continuation of diagnostic work without repeating previous steps.

### 2.3 Integrated network visualization

The pathway network module uses Cytoscape.js (Franz *et al.* 2016) to visualize interactions between genes identified in the patient’s molecular data. Nodes represent genes assigned to a user-selected KEGG pathway (Kanehisa *et al.* 2016), and edges represent protein-protein interactions retrieved from STRING DB (Szklarczyk *et al.* 2023) for that gene set. STRING provides empirical, confidence-scored interactions. For gene-level pathway, disease, and drug context, users can additionally consult external knowledge resources such as KEGG MEDICUS (Kanehisa *et al.* 2016). Nodes are colored according to log2 fold-change values from gene expression data, visually highlighting upregulated and downregulated genes. The platform supports highlighting of oncogenic and pathogenic variants as well as fusion gene partners identified and flagged in previous modules, revealing how genomic alterations affect pathway architecture. The network visualization enables users to adjust interaction confidence thresholds to balance network comprehensiveness with reliability, filtering interactions based on the estimated probability that they represent true biological associations. Evidence type filtering allows refinement of displayed connections by selecting specific types of supporting evidence, including experimental data, curated pathway databases, co-expression patterns, and text-mining sources. The network can be interactively manipulated by adding or removing nodes to explore relevant biological contexts. This integrative view is particularly valuable for evaluating alterations in transcription factors and regulatory genes. Clinical geneticists can assess whether mutations and fusions affect shared pathways and identify systematic transcriptional changes that extend beyond the directly altered genes.

### 2.4 Summary and report

The summary provides a comprehensive overview of user-selected oncogenic and pathogenic variants, true-positive fusions, and deregulated genes across all datasets. To accommodate diverse reporting requirements across clinical settings, the platform supports both predefined and custom user-uploaded templates with keyword-based variable substitution, enabling clinical geneticists to maintain established formats while benefiting from automated integration of molecular findings into structured diagnostic documents.

## 3. Results

We present SeqUIaSCOPE, an open-source R Shiny platform that uniquely combines case-centric multi-omics integration with secure local and cluster-based deployment for clinical oncology diagnostics. Unlike cohort-based research platforms designed for population-level analyses, SeqUIaSCOPE is specifically architected for single-patient diagnostic workflows, integrating somatic and germline variants, fusion events, and expression profiles within a cohesive platform. By integrating research-grade multi-omics analysis with practical clinical workflow features, SeqUIaSCOPE bridges the gap between cohort-based research platforms and the operational demands of routine diagnostic practice. Beyond being free and open-source, SeqUIaSCOPE offers several practical advantages. All analysis stays on local or institutional infrastructure, so sensitive data never leave the institution. The code is fully auditable and reporting templates are customizable, without vendor lock-in. Deployment through Docker and Kubernetes requires no per-seat licensing.

### 3.1 Development and validation

SeqUIaSCOPE is an interpretation-support tool. It integrates and displays molecular findings for expert review, while the diagnostic conclusion remains with the clinical geneticist. Its evaluation therefore addressed fitness for routine diagnostic use rather than diagnostic accuracy. Requirements were defined iteratively with clinical geneticists. The platform was evaluated on previously diagnosed cases, comprising 20 high-risk pediatric solid tumors and 30 hereditary cancer syndrome germline cases. For these cases, geneticists confirmed that it correctly integrated and displayed the alterations underlying the established diagnoses within a single interface. Since then, the platform has been used in the routine assessment of 15 further patients. Case-level detail is not reported, as these are sensitive patient data.

## Supplementary Material

btag630_Supplementary_Data
