# Supplementary material for: SeqUIaSCOPE: multi-omics data integration platform for single-patient clinical oncology pathway exploration
Source: Bioinformatics. 2026 Aug 20;42(9):btag630. doi: 10.1093/bioinformatics/btag630 (PMC13537515; doi:10.1093/bioinformatics/btag630)
Supplement: Supplementary file 1 [file btag630_supplementary_data.pdf]

SeqUIaSCOPE Documentation

Kateřina Jurásková

2026-07-21

- Introduction
  - Live demo
- How to install and run
- Data Requirements
  - Input Files Overview
  - File Naming Rules
  - Directory Layouts
  - Required Columns
- Upload Data
  - Step 1: Directory, Patients, and Datasets
  - Step 2: Validation
  - Fusion Pre-processing
  - Session Management
- Working with Tables
  - Default Filters
  - Sorting
  - Filtering and Column Visibility
  - Resizing Columns
  - Exporting Data
  - Flagging Items for the Report
  - Marking an Analysis as Complete
- Variant Calling
  - Filtering Variants
  - Flagging Variants
  - Somatic-Specific Visualisations
- Fusion Genes
  - Background Pre-processing
  - The Fusion Table
  - Expanded Row Preview

    - Flagging Fusions
- Expression Profile
    - The Expression Table
    - Filtering
    - Volcano Plot
    - Flagging Genes
- IGV
    - Opening IGV
    - Reference Genome
    - Bookmarks Table
    - Loading and Reloading
- Network Graph
    - Graph Elements
    - Controls
    - Subgraph
    - Data Tables
- Summary and Report
    - Overview Boxes
    - Selected Findings
    - Generating the Report
    - Custom Templates
- Configuration
    - Mounting a Custom Config
    - Config File Structure
    - Configuration Options

Introduction

SeqUIaSCOPE is an open-source web application designed for routine clinical oncology diagnostics through case-centric integration and visualization of annotated genomic variants, fusion events, and expression profiles. It includes embedded genome browsing, pathway-level interpretation, and customizable reporting. The case-centric design supports single-patient diagnosis while allowing multi-patient data management. SeqUIaSCOPE is designed for secure local or cluster-based deployment using Docker or Kubernetes, ensuring that all patient data remains within institutional infrastructure.

Live demo

We are hosting a live demo of SeqUIaSCOPE to showcase its capabilities and allow users to explore the platform in action. **Access the demo at: Live Demo (https://sequiascope.dyn.cloud.e-infra.cz/)**

Important Notes:

- Demo data are anonymized and does not represent real clinical cases
- Some features may have limited functionality due to data sensitivity requirements
- Genome browsing is available only for the fusion gene dataset, limited to one specific fusion (KMT2A::MLLT3) and the immediate vicinity of its breakpoints
- While the demo runs the full version of SeqUIaScope, uploading your own data is not supported
- For production use with your own data, deploy SeqUIaSCOPE locally using Docker or on cluster using Helm chart

How to load demo data:

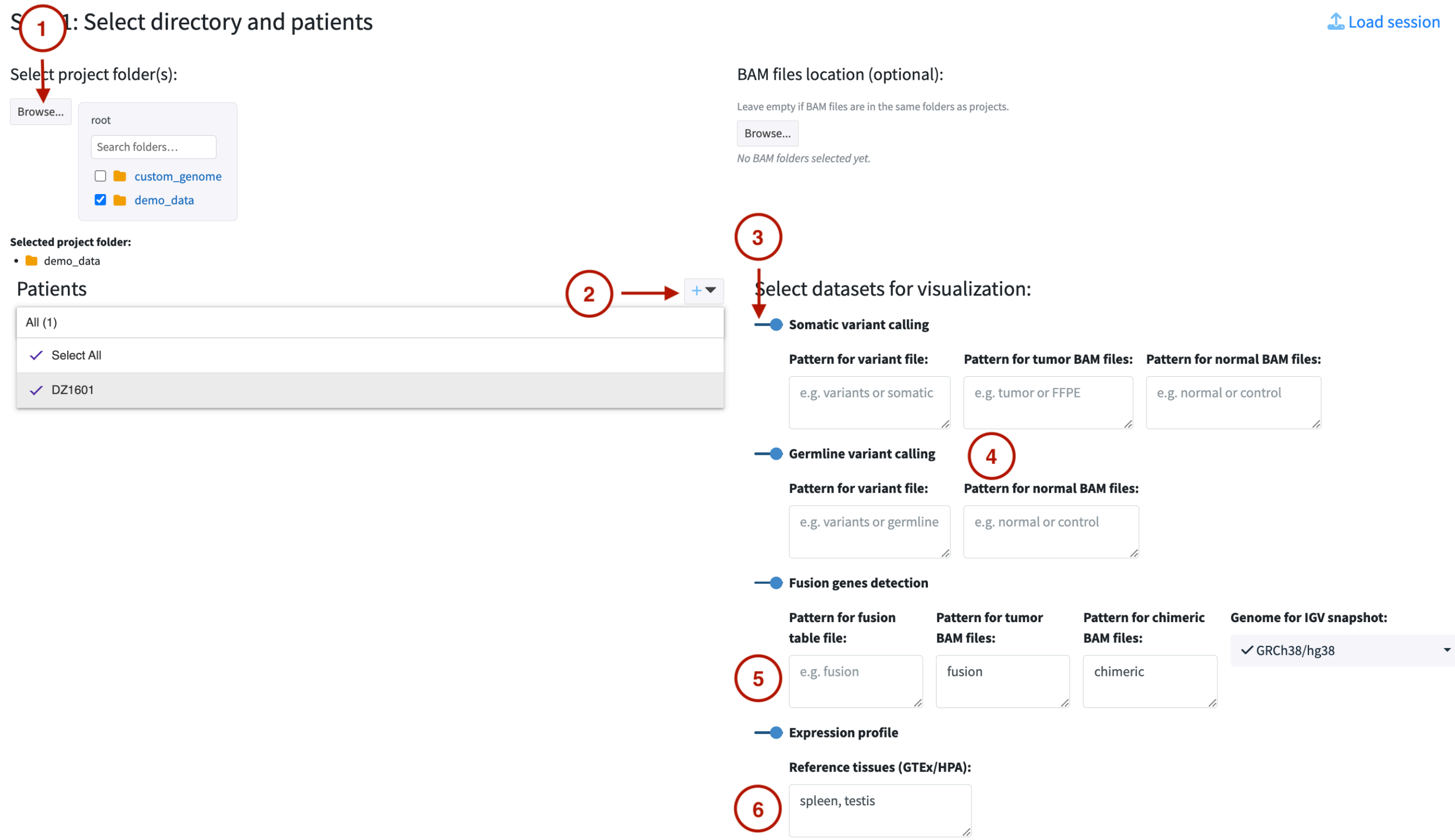

1. Select **demo_data** folder as a directory with patient data
2. Add patient name as **DZ1601**
3. Select all datasets (Somatic variant calling, Germline variant calling, Fusion gene detection and Expression profile).
4. Leave patterns for somatic and germline variants empty.
5. Fill fusion detection pattern for tumor BAM file as **fusion** and for chimeric BAM file as **chimeric**. Genome for IGV snapshot should be set to GRCh38/hg38
6. Fill reference tissues for expression profile as **spleen, testis**

How to install and run

SeqUIaSCOPE can be deployed in two ways:

- **On a cluster** using Kubernetes + Helm — suitable for shared or production environments
- **Locally** using Docker Compose — suitable for single-machine use

Full installation and setup instructions are available in the README on GitHub (https://github.com/BioIT-CEITEC/sequiascope).

Data Requirements

SeqUIaSCOPE accepts four types of genomic data: somatic variant calling results, germline variant calling results, fusion gene detection results, and gene expression profiles. Not all four are required — you can upload any combination depending on your analysis needs.

Before uploading, make sure your files follow the naming and format rules described on this page. The application automatically scans your directory for files and uses these rules to identify which file belongs to which patient and dataset type. Errors at upload time are almost always caused by naming issues.

Input Files Overview

☐ Somatic Variant Calling

REQUIRED

- ☐ `.tsv` — annotated somatic variants

OPTIONAL

- ☐ Tumor DNA `.bam` + `.bai` — for IGV
- ☐ Normal DNA `.bam` + `.bai` — for IGV
- ☐ `mutation_loads.tsv` — tumour mutational burden

☐ Germline Variant Calling

REQUIRED

- ☐ `.tsv` — annotated germline variants

OPTIONAL

- ☐ Normal DNA `.bam` + `.bai` — for IGV

☐ Fusion Gene Detection

REQUIRED

- ☐ `.tsv` or `.xlsx` — combined Arriba + STARFusion results

OPTIONAL

- ☐ Tumor RNA `.bam` + `.bai` — for IGV snapshots
- ☐ Chimeric `.bam` + `.bai` — for IGV snapshots
- ☐ Arriba `.pdf` + `.tsv` — for expanded row preview

☐ Expression Profile

REQUIRED

- ☐ `.tsv` or `.xlsx` — gene expression data

OPTIONAL

- ☐ One file per tissue if comparing multiple reference tissues

Tip: When you have a choice, always prefer `.tsv` over `.xlsx`. TSV files load faster and are less likely to cause formatting issues.

Fusion gene detection requires results from both **Arriba** and **STARFusion** as upstream tools. The required input file is a pre-processed table combining the output of both callers. The Arriba `.pdf` and `.tsv` output files are separate and optional — they unlock the expanded row preview in the Fusion Genes module.

File Naming Rules

SeqUIaSCOPE identifies files by scanning the full file path (folder names + file name together). It looks for specific **keywords** anywhere in the path to decide what type each file is. This means a keyword can appear in the filename itself **or** in a parent folder name — both work.

Keyword Reference

File type	Path must contain	Path must NOT contain
Somatic variant file	`somatic`	—
Germline variant file	`germline`	—
Fusion gene file	`fusion` or `fuze`	`arriba`, `STAR`
Tumor RNA BAM	user-defined pattern (set during upload)	`Chimeric`, `transcriptome`
Chimeric BAM	user-defined pattern (set during upload)	`transcriptome`
Arriba output files	`arriba`	`discarded`, `STAR`
Expression file	`expression` or `RNAseq`	`genes_of_interest`
TMB file	`somatic`, and the filename must end with `mutation_loads`	—

The keyword can live anywhere in the path — these two examples both work:

```
project_root/patient_001/somatic_variants.tsv    # keyword in filename
project_root/somatic_data/patient_001/variants.tsv  # keyword in folder name
```

Patient ID in path: Every per-patient file must have the patient ID somewhere in its full path (either in the filename or a parent folder). Files without the patient ID will not be matched. The TMB file (`mutation_loads`) and the Genes of Interest file are the only exceptions — they are shared across all patients and do not need a patient ID in the path.

BAM Files

BAM files don't use the keyword system. Instead, you define **patterns** during upload (e.g. `tumor`, `FFPE`, `chimeric`) and the application finds BAM files whose names match those patterns. See Upload Data — Step 1 for details.

For every `.bam` file, a corresponding index file must exist in the same directory, named either `file.bam.bai` or `file.bai`.

Arriba Output Files

If you provide Arriba output files, both the `.pdf` and `.tsv` must be present as a matched pair (same base name, different extension). A `.pdf` without a matching `.tsv` will be ignored, and vice versa.

Expression Files with Multiple Tissues

When comparing against multiple reference tissues, provide one file per tissue. The tissue name must appear in the filename (e.g. `blood_expression.tsv`, `liver.tsv`). Use underscores instead of spaces — `blood_vessel.tsv`, not `blood vessel.tsv`.

Tissue names you enter in the upload form must match the names in the filenames. If no tissue names are provided, the application looks for a single expression file per patient containing `expression` or `RNAseq` in its path.

Directory Layouts

SeqUIaSCOPE is flexible — your data can be organised in many different ways as long as the naming rules above are respected. Below are three common layouts that all work correctly. The key principle is that the **patient ID** and **dataset keyword** must both appear somewhere in each file's path.

> **Choose your root directory carefully.** In the upload form you select a single root directory that contains all your patient data. Pick the most specific folder that still contains everything — selecting a very broad directory (e.g. your home folder) may cause the scanner to pick up unrelated files and confuse file matching.

Option A — one folder per data type

Best when your pipeline already separates outputs by analysis type.

```
project_root/                          ← select this as root
├── somatic_data/
│   ├── mutation_loads.tsv
│   ├── patient_001/
│   │   ├── somatic_variants.tsv
│   │   ├── tumor.bam  +  tumor.bam.bai
│   │   └── normal.bam  +  normal.bam.bai
│   └── patient_002/ ...
├── germline_data/
│   ├── patient_001/
│   │   ├── germline_variants.tsv
│   │   └── normal.bam  +  normal.bam.bai
│   └── patient_002/ ...
├── fusion_data/
│   ├── patient_001/
│   │   ├── fusions.tsv
│   │   ├── fusion.bam  +  fusion.bam.bai
│   │   ├── chimeric.bam  +  chimeric.bam.bai
│   │   ├── arriba_report.pdf
│   │   └── arriba_results.tsv
│   └── patient_002/ ...
└── expression_data/
    ├── patient_001/
    │   └── expression.tsv
    └── patient_002/ ...
```

Option B — BAM files stored separately from analysis results

Common when primary (alignment) and secondary (variant/fusion calling) outputs live in separate trees.

```
project_root/                           ← select this as root
├── alignments/
│   ├── DNA/
│   │   ├── patient_001/
│   │   │   ├── tumor.bam  +  tumor.bam.bai
│   │   │   └── normal.bam  +  normal.bam.bai
│   │   └── patient_002/ ...
│   └── RNA/
│       ├── patient_001/
│       │   ├── fusion.bam  +  fusion.bam.bai
│       │   └── chimeric.bam  +  chimeric.bam.bai
│       └── patient_002/ ...
└── results/
    ├── somatic_data/
    │   ├── mutation_loads.tsv
    │   ├── patient_001/ somatic_variants.tsv
    │   └── patient_002/ ...
    ├── germline_data/
    │   ├── patient_001/ germline_variants.tsv
    │   └── patient_002/ ...
    ├── fusion_data/
    │   ├── patient_001/
    │   │   ├── fusions.tsv
    │   │   ├── arriba_report.pdf
    │   │   └── arriba_results.tsv
    │   └── patient_002/ ...
    └── expression_data/
        ├── patient_001/ expression.tsv
        └── patient_002/ ...
```

Option C — all files flat per patient

Works for smaller projects or when a single pipeline writes everything into one folder per patient.

```
project_root/                            ← select this as root
├── somatic_mutation_loads.tsv            ← shared in root, "somatic" must be in the name (in Options A and B the folder already provides it)
├── patient_001/
│   ├── somatic_variants.tsv
│   ├── germline_variants.tsv
│   ├── fusions.tsv
│   ├── tumor.bam  +  tumor.bam.bai
│   ├── normal.bam  +  normal.bam.bai
│   ├── fusion.bam  +  fusion.bam.bai
│   ├── chimeric.bam  +  chimeric.bam.bai
│   ├── arriba_report.pdf
│   ├── arriba_results.tsv
│   └── expression.tsv
└── patient_002/ ...
```

Required Columns

Each data file must contain a set of required columns. Column names are matched case-insensitively, so their capitalization does not need to match exactly. Any additional columns in your file are allowed. They will appear in the table but without custom formatting or labels.

Somatic Variant File

Column	Type	Description
`var_name`	string	Variant identifier
`gene_symbol`	string	Gene symbol
`tumor_variant_freq`	numeric	Variant allele frequency in tumour
`tumor_depth`	integer	Sequencing depth at variant position in tumour
`gene_region`	string	Genomic region (e.g. `exon`, `intron`, `splice`)
`gnomAD_NFE`	numeric	gnomAD Non-Finnish European allele frequency
`consequence`	string	Variant consequence (e.g. `missense_variant`, `stop_gained`)
`HGVSc`	string	HGVS coding sequence notation
`HGVSp`	string	HGVS protein sequence notation
`variant_type`	string	Variant type (e.g. `SNV`, `insertion`, `deletion`)
`all_full_annot_name`	string	Full annotation name

Optional columns recognised with custom labels:

Column	Description
`in_library`	Number of times `var_name` was observed in the project cohort (added automatically if absent)
`clinvar_sig`	ClinVar clinical significance
`clinvar_DBN`	ClinVar disease name
`CGC_Somatic`	Cancer Gene Census somatic annotation
`fOne`	fOne database annotation
`COSMIC`	COSMIC database annotation
`HGMD`	HGMD database annotation
`snpDB`	dbSNP annotation

TMB File (`mutation_loads`)

Column	Type	Description
`patient`	string	Patient ID — must match the IDs entered in the upload form
`TMB`	numeric	Tumour mutational burden value

patient	TMB
P001	0.17
P002	0.74

Germline Variant File

Column	Type	Description
`var_name`	string	Variant identifier
`gene_symbol`	string	Gene symbol
`variant_freq`	numeric	Variant allele frequency
`coverage_depth`	integer	Sequencing depth at variant position
`gene_region`	string	Genomic region (e.g. `exon`, `intron`, `splice`)
`gnomAD_NFE`	numeric	gnomAD Non-Finnish European allele frequency
`clinvar_sig`	string	ClinVar clinical significance
`consequence`	string	Variant consequence
`HGVSc`	string	HGVS coding sequence notation
`HGVSp`	string	HGVS protein sequence notation
`variant_type`	string	Variant type (e.g. `SNV`, `insertion`, `deletion`)
`all_full_annot_name`	string	Full annotation name

Optional columns:

Column	Description
`in_library`	Number of times `var_name` was observed in the project cohort (added automatically if absent)
`clinvar_DBN`	ClinVar disease name
`CGC_Germline`	Cancer Gene Census germline annotation
`trusight_genes`	TruSight gene panel annotation
`fOne`	fOne database annotation
`snpDB`	dbSNP annotation

GnomAD column is the population allele frequency that SeqUIaSCOPE uses to filter variants by how common they are. The preferred reference is `gnomAD_NFE`. If it is not present, the application falls back to the first available column from a priority list and uses it as the main frequency reference for filtering. Column matching is case insensitive. From gnomAD it accepts `gnomAD_NFE`, `gnomAD_AFR`, `gnomAD_AMR`, `gnomAD_EAS`, `gnomAD_SAS`, `gnomAD_FIN`, `gnomAD_ASJ`, `gnomAD_MID`, and the overall `gnomAD_AF`. From 1000 Genomes it accepts `1000g_eur_af`, `1000g_amr_af`, `1000g_afr_af`, `1000g_eas_af`, `1000g_sas_af`, and the overall `1000g_af`. Your file needs at least one of these, and the filter label in the module adjusts to whichever column was found.

Fusion Gene File

This is a pre-processed file combining results from both **Arriba** and **STARFusion**. Column names `chrom1`/`chrom2` are accepted as alternatives to `chr1`/`chr2` and will be renamed automatically.

Column	Type	Description
`gene1`	string	First fusion partner gene name
`gene2`	string	Second fusion partner gene name
`chr1` (or `chrom1`)	string	Chromosome of first partner (with `chr` prefix, e.g. `chr2`)
`chr2` (or `chrom2`)	string	Chromosome of second partner
`pos1`	numeric	Genomic position of first partner breakpoint
`pos2`	numeric	Genomic position of second partner breakpoint
`strand1`	string	Strand orientation of first partner (`+` or `-`)
`strand2`	string	Strand orientation of second partner (`+` or `-`)
`arriba.called`	boolean	Whether Arriba called this fusion
`starfus.called`	boolean	Whether STARFusion called this fusion
`overall_support`	numeric	Overall read support for the fusion
`arriba.confidence`	string	Arriba confidence level (`high`, `medium`, or `low`)
`arriba.site1`	string	Breakpoint site annotation for first partner
`arriba.site2`	string	Breakpoint site annotation for second partner

Optional columns:

Column	Description
`DB_count`	Number of databases listing this fusion
`DB_list`	Database names
`arriba.reading_frame`	Reading frame of the fusion predicted by Arriba (added automatically if absent)
`arriba.split_reads`	Split reads supporting the fusion (Arriba)
`arriba.discordant_mates`	Discordant mate pairs (Arriba)
`arriba.break_coverage`	Coverage at first breakpoint (Arriba)
`arriba.break2_coverage`	Coverage at second breakpoint (Arriba)
`arriba.break_seq`	Sequence at breakpoint (Arriba)
`starfus.split_reads`	Split reads supporting the fusion (STARFusion)
`starfus.discordant_mates`	Discordant mate pairs (STARFusion)
`starfus.counter_fusion1`	Counter fusion reads for first gene (STARFusion)
`starfus.counter_fusion2`	Counter fusion reads for second gene (STARFusion)
`starfus.splice_type`	Splice junction type (STARFusion)
`starfus.break_seq`	Breakpoint sequence (STARFusion)

Expression Profile File

Column	Type	Description
`sample`	string	Sample ID
`feature_name`	string	Gene name
`geneid`	string	Gene ID (Ensembl, RefSeq, or other)
`all_kegg_gene_names`	string	KEGG gene names
`log2FC`	numeric	Log2 fold change (tumour vs. reference)
`p_value`	numeric	P-value
`p_adj`	numeric	Adjusted p-value

Optional columns:

Column	Description
`pathway`	Pathway name (retrieved automatically from KEGG if absent)
`refseq_id`	RefSeq gene ID
`type`	Gene type
`gene_definition`	Gene description
`num_of_paths`	Number of pathways the gene participates in

Genes of Interest (GOI)

SeqUIaSCOPE ships with a built-in genes of interest list used for expression highlighting and network visualisation. To use a custom list instead, provide a `.tsv` or `.xlsx` file and update the path in `reference_paths.json` (see Configuration).

Column	Required	Description
`gene`	✅	Gene name
`pathway`	optional	Pathway name — retrieved from KEGG automatically if absent

If the `pathway` column is provided, your own pathway classifications are used instead of KEGG.

gene	pathway
BRCA1	DNA damage/repair
TP53	RTK Signalling

Upload Data

The Upload Data module is the starting point of every analysis session. It guides you through selecting your data directory, specifying patients and datasets, and validating that all required files have been found before the analysis begins.

If you have previously saved a session, you can skip this process entirely by loading it directly — see Session Management below.

Step 1: Directory, Patients, and Datasets

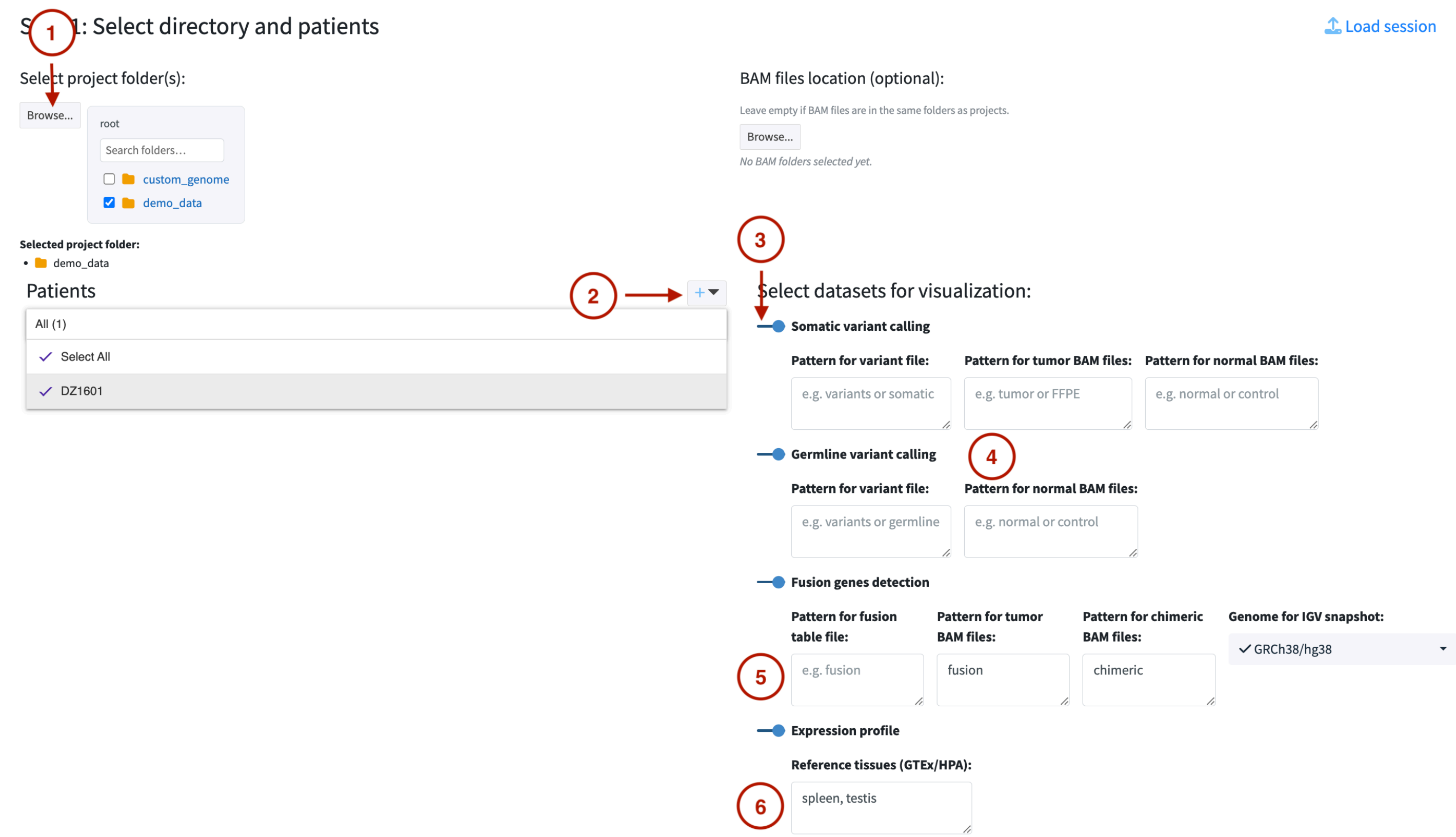

Select directory

Click **Browse** to select the root directory containing your patient data. The file browser opens at `/input_files` by default — this is the directory mounted into the application when running via Docker. If you are running the application locally without Docker, it opens at the working directory.

Choose the most specific directory that still contains all patient folders. Selecting a too-broad directory (e.g. your home folder) may cause the file scanner to pick up unrelated files. See Data Requirements — Directory Layouts for guidance.

Use the optional **BAM files location** field only when your alignment files are in a separate directory from your analysis results, as in Option B of the directory layouts. Leave it empty when BAM files are in the same patient folders as the rest of the data. When set, this directory becomes the source for BAM files and any BAM files inside the project folders are ignored. Patient IDs and BAM patterns still apply.

Add patients

Enter one or more patient IDs using the **+** button next to the Patients panel. You can type multiple IDs at once, separated by commas or new lines. Patient IDs must match the identifiers used in your file paths — the scanner uses them to match files to the correct patient.

Select datasets

Toggle on the datasets you want to analyse. For each selected dataset, additional options appear:

Somatic variant calling

- *Pattern for variant file* — an optional string that appears in the variant filename, used to distinguish the annotated variant file from other `.tsv` files in the same directory (e.g. if your pipeline produces both `DZ1601.variants.tsv` and `DZ1601.final.variants.tsv`, you can use a pattern to select only the correct one). Leave empty if there is no ambiguity.
- *Pattern for tumor BAM files* — a string that appears in the tumor BAM filename (e.g. `tumor`, `FFPE`). Leave empty if you do not want to use BAM files for this dataset. Enter `none` if BAM files are available but have no prefix beyond the patient ID (e.g. `DZ1601.bam` instead of `DZ1601_tumor.bam`).
- *Pattern for normal BAM files* — same logic as above for normal DNA BAM files.

Germline variant calling

- *Pattern for variant file* — same as for somatic variants.
- *Pattern for normal BAM files* — for normal DNA BAM files.

Fusion gene detection

- *Pattern for tumor BAM files* — for tumor RNA BAM files.
- *Pattern for chimeric BAM files* — for chimeric BAM files (e.g. `chimeric`).
- *Genome for IGV snapshot* — select the reference genome to use when generating IGV snapshots of fusion breakpoints. Available options: `GRCh38/hg38`, `hg38 1kg/GATK`, `GRCh37/hg19`, `T2T CHM13-v2.0/hs1`, `Custom` (configured via `reference_paths.json`), or **Don't create IGV snapshots** if you want to skip IGV snapshot generation.

Expression profile

- *Reference tissues* — enter tissue names matching the tissue identifiers in your expression filenames (e.g. `blood`, `liver`, `blood_vessel`). If your data contains a single expression file

per patient with no tissue suffix, leave this field empty. Different patients can have different tissue sets — tissues that cannot be matched to a file for a given patient are simply treated as unavailable for that patient.

> **BAM pattern matching:** Patterns are matched against the filename. A pattern of `tumor` will match `patient_001_tumor.bam` but not `patient_001_normal.bam`. Multiple patterns can be entered separated by commas (e.g. `tumor, FFPE`). Leave the field empty to skip BAM files entirely. Enter `none` if your BAM files have no pattern beyond the patient ID.

> **Tissue names:** Spaces are not supported — use underscores, dots, or hyphens as separators (e.g. `blood_vessel`, `blood.vessel`, or `blood-vessel`). The tissue name must appear somewhere in the expression filename — see Data Requirements — Expression Files with Multiple Tissues.

Click **Next** to proceed to validation.

Step 2: Validation

After clicking Next, the application scans your selected directory and attempts to match files to each patient and dataset. The results are displayed in a validation table.

Each file type is shown with a status icon:

- 🟢 **Green** — required file found, everything is in order
- 🟠 **Orange** — optional files are missing (e.g. BAM files for IGV). The analysis can proceed, but some features will be unavailable
- 🔴 **Red** — a required file is missing or multiple conflicting files were found. This must be resolved before you can continue

Review the table carefully, especially the tissue-to-file pairing for expression data if you specified multiple tissues. Once you are satisfied, click **Confirm** to load the data and proceed to the analysis modules.

> **Made a mistake in Step 1?** Use the **Back to Selection** button to return and adjust your configuration without losing your patient list or dataset selections.

Files added or renamed after scanning? Use the **Refresh** button to re-scan the directory without going back to Step 1. This is useful if you noticed a missing file and added it to the directory while the validation table was open.

Common errors and solutions

Required file missing

The expected variant, fusion, or expression file was not found for a patient. Check that:

- The file exists in the patient directory
- The patient ID appears somewhere in the file path
- The dataset type keyword appears somewhere in the file path (see Data Requirements — Dataset Type Keywords)
- The file has the correct extension (`.tsv` for variants, `.tsv` or `.xlsx` for fusion and expression)

Multiple files found

More than one file of the same type was found for a patient. Check that:

- There are no duplicate files in the directory
- BAM file patterns are specific enough to select only one file per type

Missing BAM or BAI

A BAM file was found but its index file is missing. Generate the index with:

```
samtools index file.bam
```

The index must be named `file.bam.bai` or `file.bai` and placed in the same directory as the BAM.

Missing Arriba PDF or TSV

Only one of the two Arriba output files was found. Both the `.pdf` and the `.tsv` must be present with matching base names and both must contain `arriba` in their path.

Tissue matching error

The number of expression files found does not match the number of tissues specified, or the tissue names cannot be matched to file names. Check that:

- Each tissue has exactly one corresponding expression file
- Tissue names in the form match the tissue identifiers in the filenames
- Underscores are used instead of spaces in multi-word tissue names

Fusion Pre-processing

When fusion gene data is confirmed and BAM files are available, the application automatically starts a background pre-processing step before the Fusion Genes module becomes available. This step generates IGV snapshots of each fusion breakpoint and extracts Arriba report images. Tasks that can take several minutes depending on the number of fusions and the size of BAM files.

Crucially, this runs **in the background** — you do not need to wait for it to finish. You can immediately start exploring somatic variants, germline variants, and expression data in the other modules while snapshot generation is in progress. The Fusion Genes module will progressively update as snapshots become available.

If the application detects that snapshots from a previous session already exist for the same dataset, a dialog will ask whether you want to **Resume** (reuse existing snapshots and only generate the missing ones) or **Clean Start** (delete existing outputs and start fresh). This is useful if a previous session was interrupted partway through.

> If you selected *Don't create IGV snapshots* in Step 1, the IGV snapshot generation is skipped but Arriba report images are still extracted if Arriba files are available. The expanded row preview in the Fusion Genes module will contain Arriba visualisations but no IGV images.

Session Management

SeqUIaSCOPE supports saving and loading analysis sessions so that diagnostic work can be interrupted and resumed at any point without repeating the upload and validation steps.

Saving a session — click the ☐ icon in the top navigation bar at any point during your analysis. The session file captures your current configuration, selected patients and datasets, all flagged variants, fusions, and genes, any notes you have added, and the completion state of each dataset. The file is saved to the `output_files/` directory.

Loading a session — click the **Load session** button in the top right corner of Step 1. Select the previously saved session file. The application will restore your configuration and all selections, skipping the directory selection and validation steps.

> **Accidental navigation:** If you try to close or refresh the browser tab after data has been loaded, the application will show a warning asking you to confirm. This is to prevent accidental loss of unsaved work.

Working with Tables

All data in SeqUIaSCOPE — variants, fusions, and expression results — is displayed using interactive tables built with the `reactable` package. This page describes the features common to all tables. You do not need to read it before exploring the modules, but it is worth coming back to if something in a table behaves unexpectedly.

Default Filters

When you first open a module, the table is not necessarily showing all available data. Each module applies a set of default filters on load to focus the view on the most clinically relevant entries and keep the application responsive with large datasets.

For **somatic and germline variant calling**, the default filters are the most restrictive — see Variant Calling — Default Filters for the exact criteria. For **fusion gene detection** and **expression profile**, no row filters are applied, but the column selection in the filter menu may hide some columns by default.

> **If the table appears empty or unexpectedly small**, check the active filters before assuming data is missing. The filter menu and the column filter inputs above each column header show what is currently applied.

Sorting

Click any column header to sort the table by that column. Clicking again reverses the order. A third click removes the sort.

Multi-column sorting is supported by holding `Shift` while clicking additional column headers. This sorts first by the primary column, then by each additional column in the order you clicked them. To remove a secondary sort, hold `Shift` and click the column header again until it cycles back to unsorted.

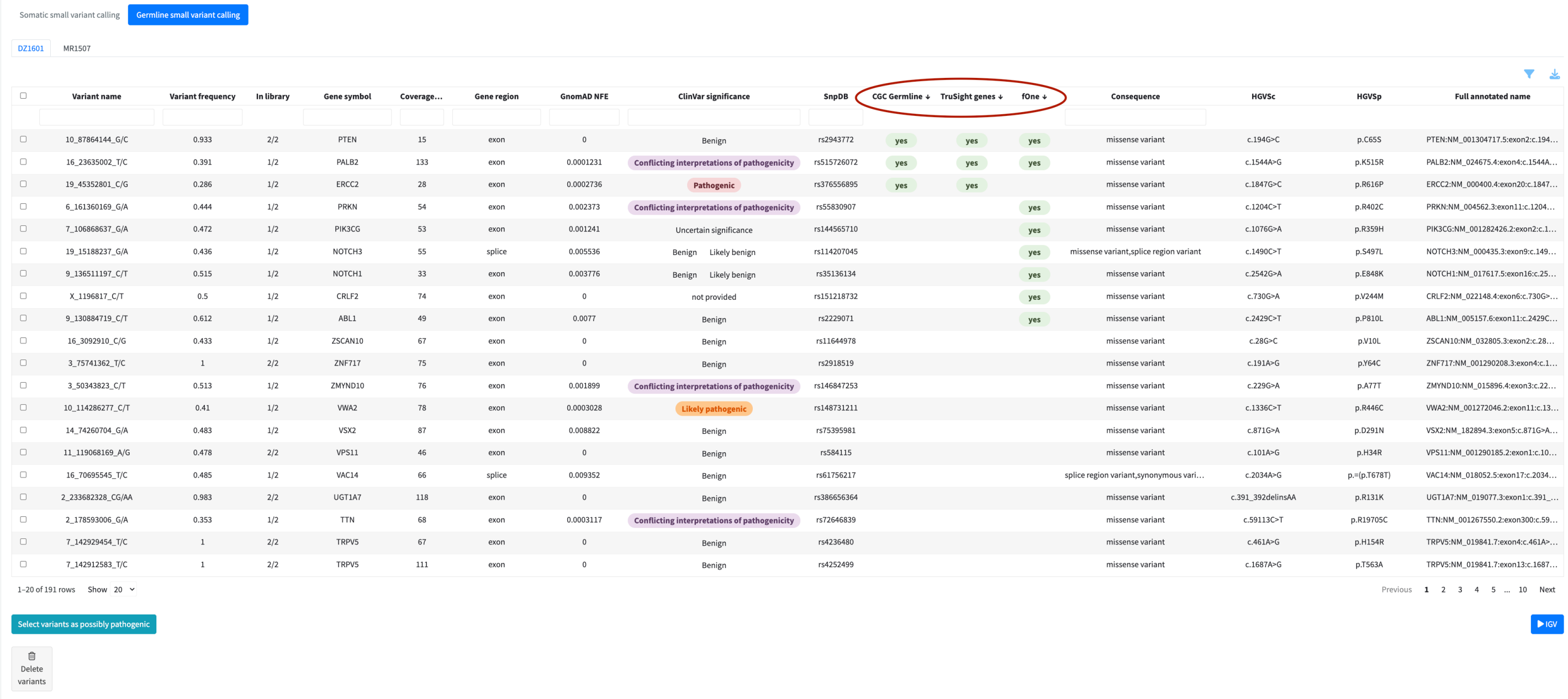

Filtering and Column Visibility

Each column has a filter input directly below the header. Typing in a filter box narrows the table to rows matching your input in that column. Filters across multiple columns are combined — a row must satisfy all active filters to remain visible.

The **filter menu** (accessible via the filter icon above the table) provides additional filtering options specific to each module, as well as a column visibility selector that lets you show or hide individual columns to focus on what matters for your analysis.

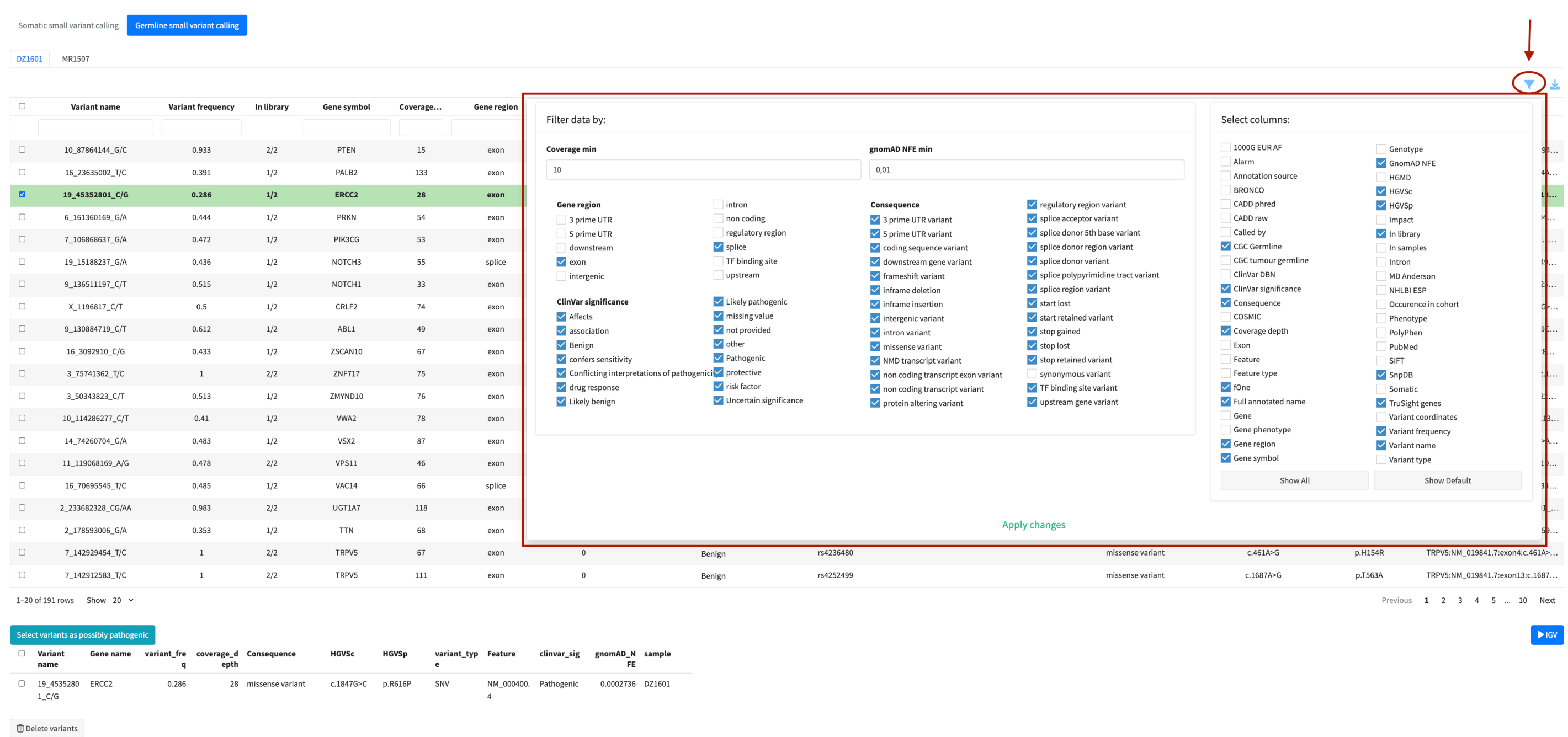

Tip: Apply filters before flagging variants or fusions for the report. Working with a filtered subset significantly reduces table reload times and keeps the application responsive.

Resizing Columns

Column widths can be adjusted by dragging the border between column headers left or right. This is available in all main data tables (variants, fusions, expression).

Exporting Data

Each table can be exported using the download button above it. Clicking it opens a small panel with two dropdowns. The first selects the data scope, either all data or only the rows currently visible after applying your filters. The second selects the file format from CSV, TSV, or Excel, with TSV preselected. The Download button then saves the file.

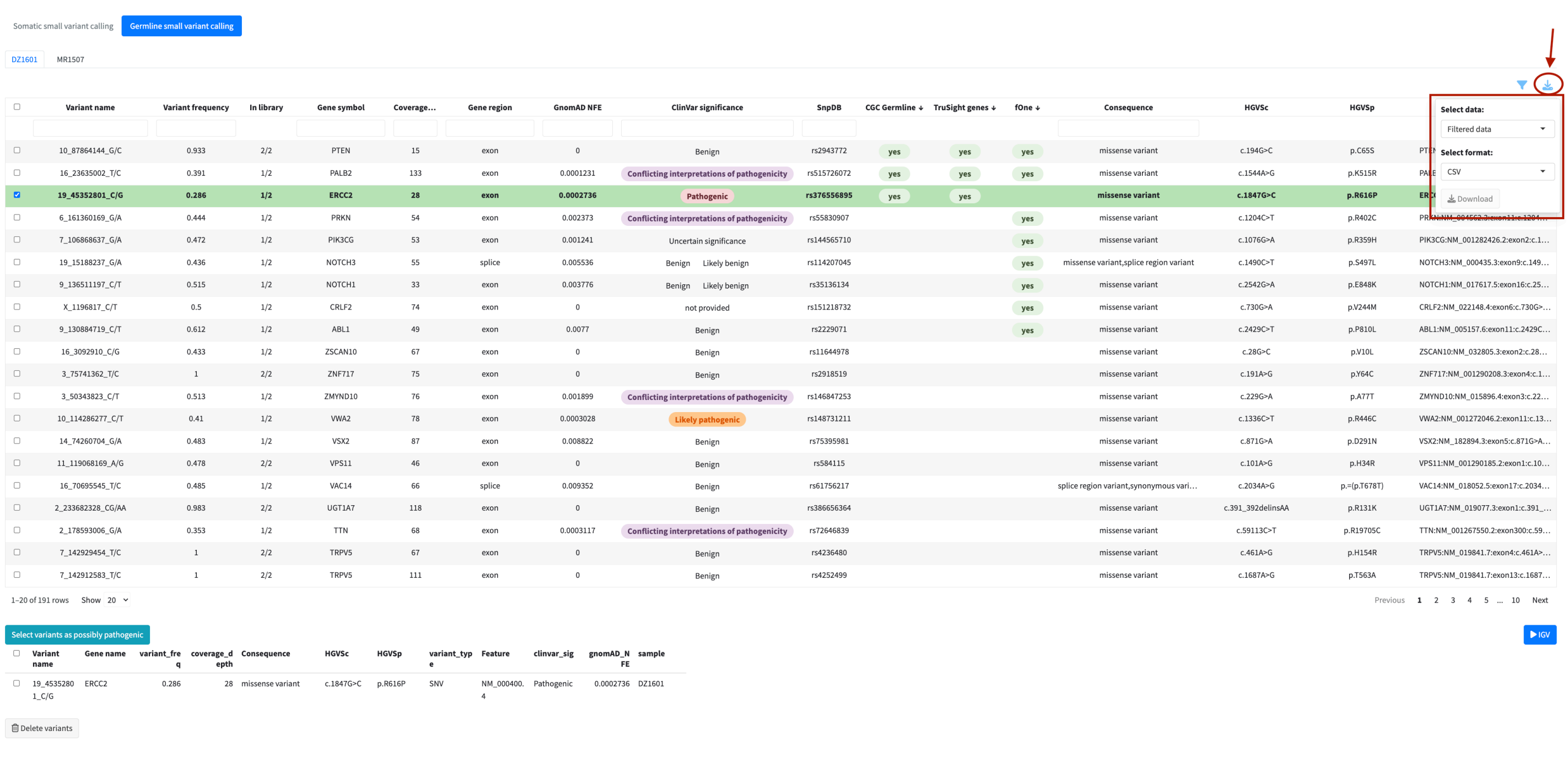

Flagging Items for the Report

for inclusion in the final clinical report. Flagged items appear in a dedicated review panel below the table where you can check your selection and remove items if needed.

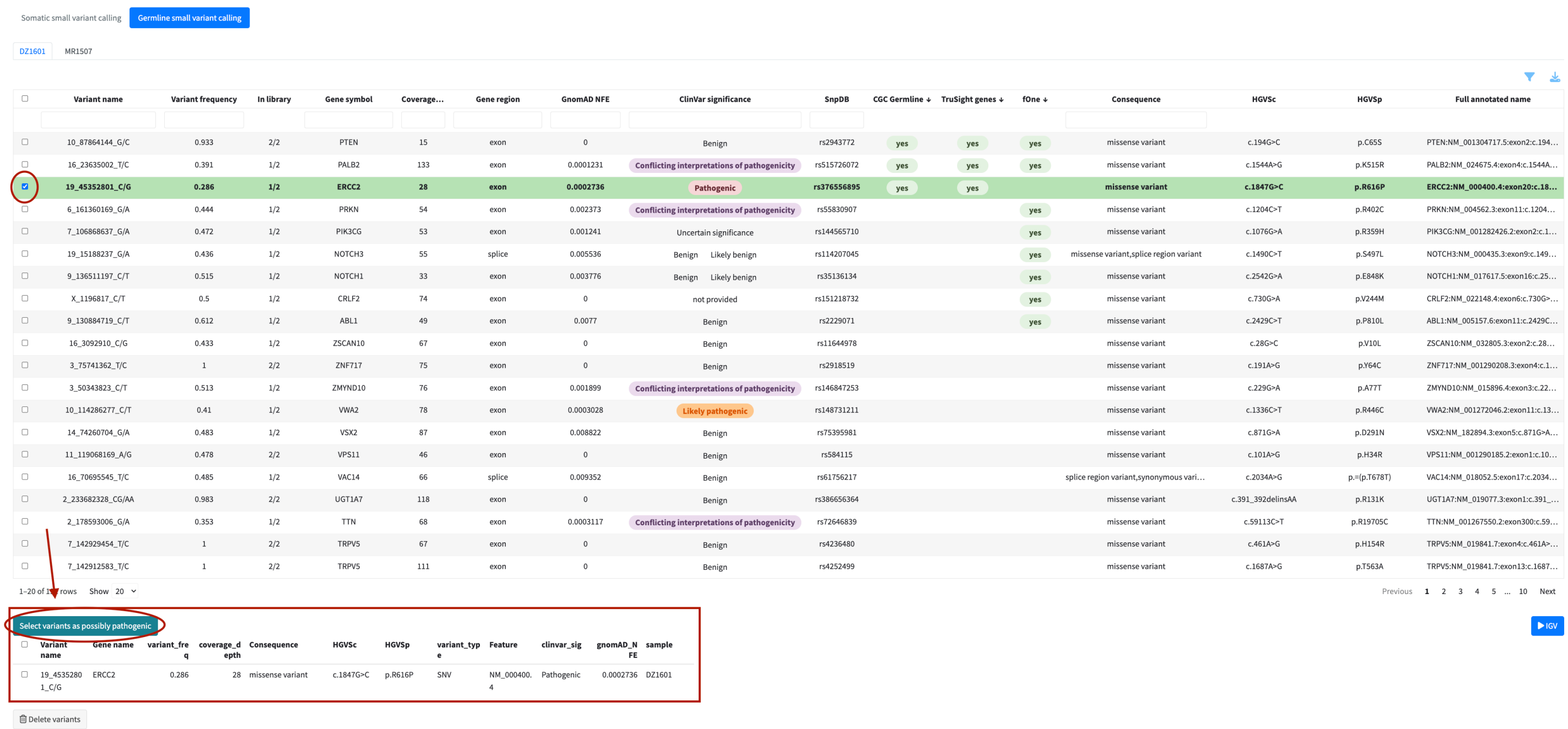

In the variant calling and fusion modules, flagged items also become available for inspection in the IGV genome browser within the same module.

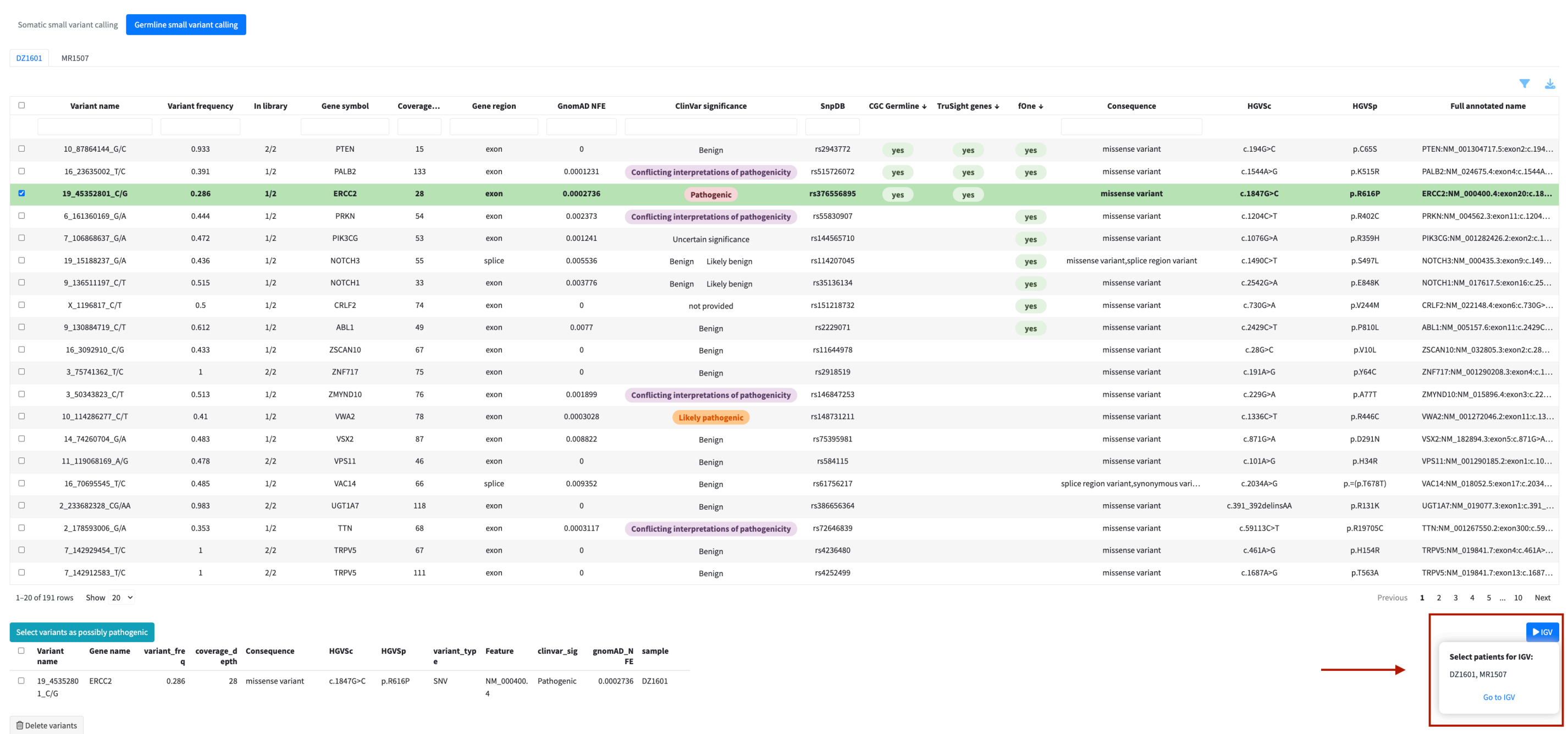

Only flagged items are included in the final report.

Marking an Analysis as Complete

Each analysis module has a switch labeled **Mark analysis complete** in the top left of the table header. It records whether you have finished reviewing that dataset for the current patient. The switch has two positions, in progress and complete, so you can see which datasets still need attention and which are done.

Beneath the Select button there is a short reminder pointing back to the switch, so once you finish flagging you know where to mark the dataset as reviewed.

In the expression module the switch sits above the two tabs rather than inside them, because completion applies to the whole expression dataset at once, not to the Genes of Interest and All Genes tabs separately.

The state is stored per patient and per dataset. Marking a dataset complete also updates its box on the summary screen, described in Summary and Report.

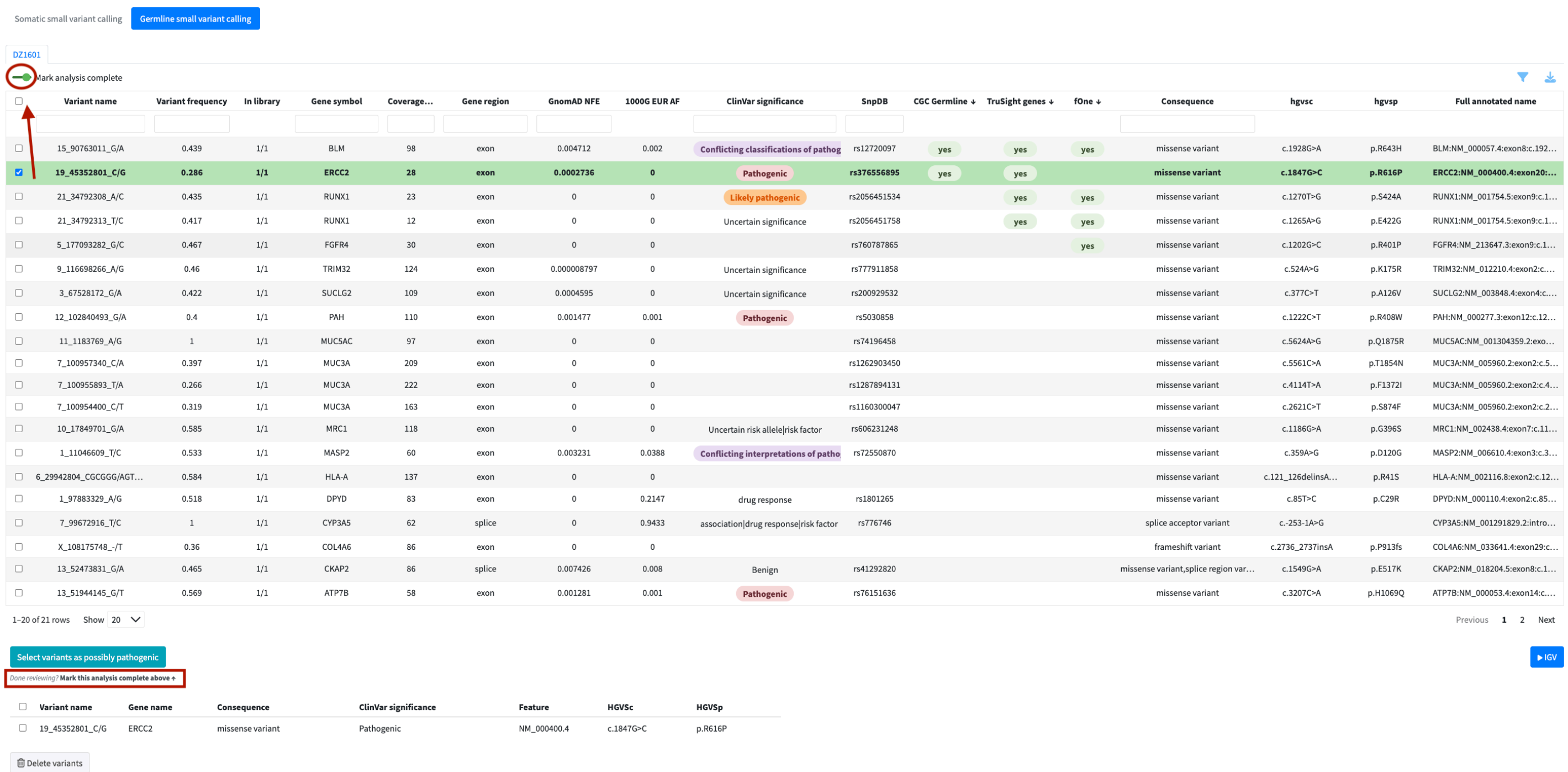

Variant Calling

The Variant Calling module is divided into two submodules: **Somatic** and **Germline** variant calling. Both provide interactive tables for reviewing, filtering, and flagging variants for the final clinical report, along with integrated IGV visualisation for read-level validation.

For general information about working with tables (sorting, filtering, column visibility, export, and flagging), see Working with Tables.

Filtering Variants

When you first open either submodule, a set of filters is applied automatically to focus the view on the most clinically relevant variants. These are not permanent — you can adjust or remove them at any time using the filter menu.

Somatic variant calling:

Filter	Default value	Description
gnomAD NFE	≤ 0.01	Rare variants only
Tumor coverage depth	> 10	Sufficient read support
Consequence	all except `synonymous variant`	Exclude silent mutations
Gene region	`exon` and `splice` only	Coding regions only

Germline variant calling:

Filter	Default value	Description
gnomAD NFE	≤ 0.01	Rare variants only (for more options, see required columns
Coverage depth	> 10	Sufficient read support
Consequence	all except `synonymous variant`	Exclude silent mutations
Gene region	`exon` and `splice` only	Coding regions only
ClinVar significance	all	Filter by clinical significance classification

> **If the table appears empty**, the most likely cause is that no variants pass the default filters for this patient. Open the filter menu and relax the gene region or gnomAD threshold to verify.

Flagging Variants

Select variants from the table and click **Select variants as possibly oncogenic** (somatic) or the equivalent button in germline to flag them for the report. Flagged variants appear in the review panel below the table, where you can also remove items from the selection.

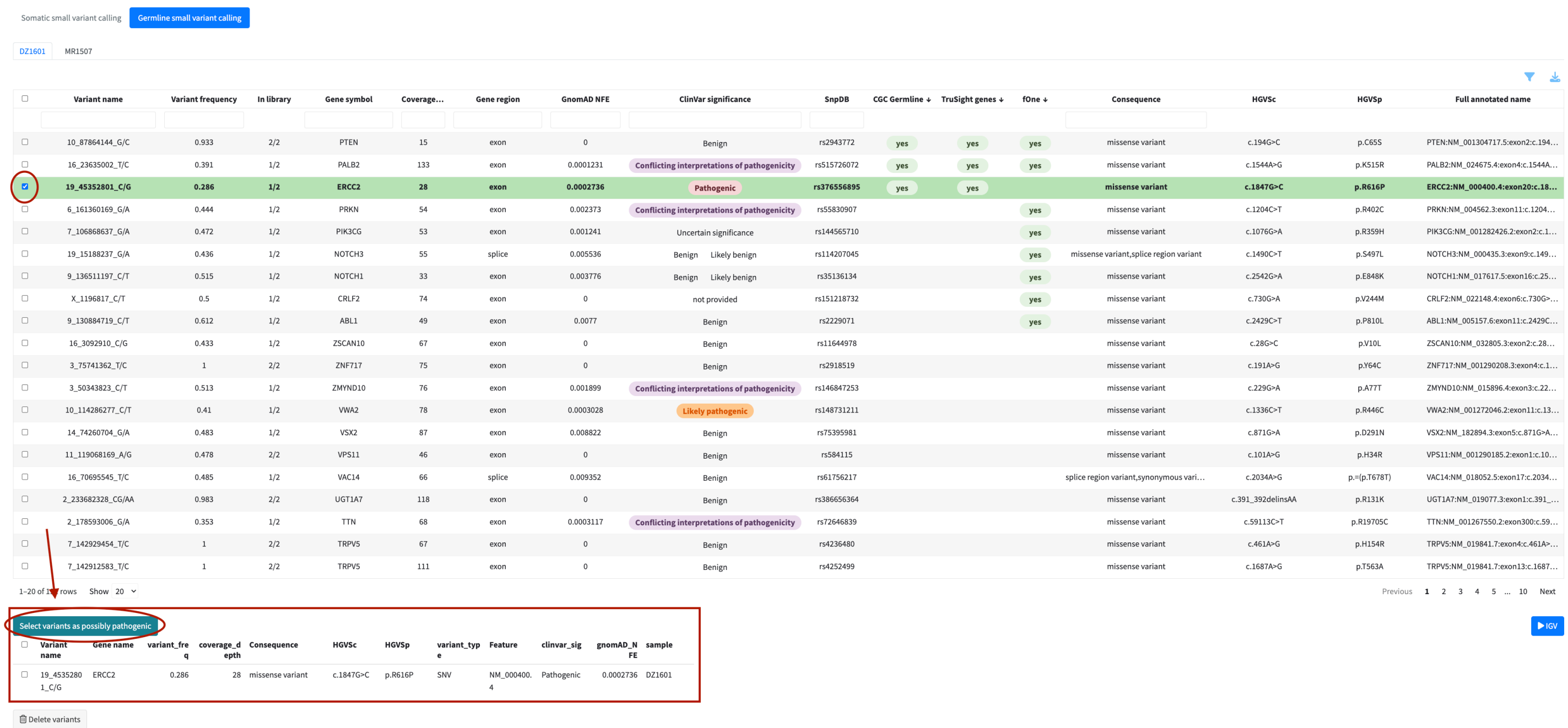

Flagged variants can be opened in the IGV viewer for read-level validation. Use the **IGV** dropdown on the right side of the module to select one or more patients (up to 4) and click **Go to IGV** to navigate to the IGV module with the selected variants pre-loaded.

IGV requires BAM files. If no BAM files were provided for this dataset during upload, the IGV dropdown will not be available.

Somatic-Specific Visualisations

The somatic submodule includes two additional visualisations, available as collapsible panels below the main table. Both can be downloaded using the export button in the top right corner of each panel.

Tumor Variant Frequency Histogram

Displays the distribution of variant allele frequencies (VAF) across all somatic variants. Flagged variants are marked as vertical lines, making it easy to see where your selected variants sit in the overall VAF distribution.

Sankey Diagram

Visualises the relationships between somatic variants, their associated genes, and the biological pathways those genes participate in. This provides a pathway-level overview of how molecular alterations are distributed across functional contexts.

Fusion Genes

The Fusion Genes module enables users to review, validate, and flag causal gene fusions for inclusion in the clinical report. It integrates supporting evidence from Arriba and STARFusion, IGV snapshots of fusion breakpoints, and Arriba visualisation reports directly within the table.

For general information about working with tables (sorting, filtering, column visibility, export, and flagging), see Working with Tables.

Background Pre-processing

Before the fusion table becomes available, the application runs a background pre-processing step that generates IGV snapshots of each fusion breakpoint and extracts images from Arriba reports. This runs in the background — you can explore other modules while it is in progress. The fusion table loads immediately and updates progressively as snapshots become available.

For more details on this step, see Upload Data — Fusion Pre-processing.

The Fusion Table

The table displays all detected fusions with columns from both Arriba and STARFusion callers. By default, the table is sorted by Arriba confidence (high first), then by whether Arriba and STARFusion both called the fusion.

Two additional columns are specific to the fusion table and are not part of the input file:

- **Visual check** — use the ✔ and ✖ buttons to mark a fusion as true positive or false positive after reviewing the evidence. This helps track which fusions have been manually reviewed without committing to flagging them for the report.
- **Notes** — a free-text field for adding case-specific observations, such as reasons for marking a fusion as false positive or notes about low coverage.

The filter menu provides column visibility control. There are no additional pre-set filters for fusion data — all fusions are shown on load.

Expanded Row Preview

Each row can be expanded to reveal a side-by-side view of the Arriba visualisation report and the IGV snapshot for that fusion. This allows quick assessment of whether a fusion is supported by sequencing evidence at both breakpoints.

The expanded row preview requires Arriba output files (`.pdf` + `.tsv`) for the Arriba report images, and BAM files for IGV snapshots. If these were not provided during upload, the expanded row will be empty or partially available.

Tip: Use the expanded row preview to quickly identify obvious false positives — for example, fusions where no signal is visible at one of the breakpoint positions in the IGV snapshot.

Flagging Fusions

Select fusions from the table and click **Select fusion as causal** to flag them for the report. Flagged fusions appear in the review panel below the table where you can also remove items from the selection.

Flagged fusions can be opened in the IGV viewer for detailed read-level inspection. Use the **IGV** dropdown to select one or more patients (up to 4) and click **Go to IGV**.

IGV requires BAM files. If no BAM files were provided during upload, the IGV dropdown will not be available.

Expression Profile

The Expression Profile module enables exploration of gene expression changes across one or more reference tissues. It provides two views: **Genes of Interest** (available when a GOI file is configured) and **All Genes**, each with its own interactive table, filter menu, and volcano plot.

For general information about working with tables (sorting, filtering, column visibility, export, and flagging), see Working with Tables.

The Expression Table

The `log2FC`, `p-value`, and `p-adj` columns are colour-coded at the cell level to highlight deregulated genes at a glance:

- Red log2FC cell — upregulated gene (`log2FC > 1`)
- Blue log2FC cell — downregulated gene (`log2FC < -1`)
- Orange p-value cell — `p-value ≤ 0.05`
- Green p-adj cell — `p-adj ≤ 0.05`

When multiple reference tissues are available, each tissue has its own set of `log2FC`, `p-value`, and `p-adj` columns, grouped under the tissue name.

Filtering

The filter menu provides the following options:

- **Tissues** — show or hide specific tissue comparisons using toggle buttons. Hidden tissues are removed from the column groups in the table.
- **Pathways** — filter rows by pathway classification using a searchable dropdown.
- **Tissue values** — for each tissue, apply quick preset filters: `log2FC > 1`, `log2FC < -1`, `p-value < 0.05`, or `p-adj < 0.05`. Multiple filters for the same tissue are combined with OR logic.
- **Column visibility** — show or hide individual columns.

Volcano Plot

Each module view includes a volcano plot available as a collapsible panel below the table. The plot shows `log2FC` on the x-axis and `-log10(p-adj)` on the y-axis.

You can adjust the plot interactively:

- **p-adj cutoff** — threshold line on the y-axis (default 0.05)
- **log2FC cutoff** — threshold lines on the x-axis (default ±1)
- **Gene labels** — number of top genes to label on the plot (default 0)

Flagging Genes

Select genes from the table and click **Select deregulated genes for report** to flag them for inclusion in the final clinical report. Flagged genes appear in the review panel below the table where you can also remove items from the selection.

IGV

The IGV module provides an embedded genome browser powered by igv.js (https://github.com/igvteam/igv.js), allowing read-level validation of flagged variants and fusion breakpoints directly within the application without any external tools.

Opening IGV

The IGV module is not opened directly from the navigation menu. Instead, it is launched from within the **Variant Calling** or **Fusion Genes** modules using the **IGV** dropdown button. Select one or more patients (up to 4) and click **Go to IGV** — the application navigates to the IGV tab with the appropriate BAM tracks and loci pre-loaded.

Only variants or fusions that have been flagged in the respective module are available for IGV inspection.

> **IGV requires BAM files.** If no BAM files were provided during upload, the IGV dropdown in the analysis modules will not be available.

Reference Genome

The reference genome is handled differently depending on which module the viewer was opened from.

When the IGV module is opened from the somatic or germline variant calling module, a genome selector is always shown before the viewer loads, so you can choose the reference for the variant tracks. The available options are `GRCh38/hg38`, `hg38 1kg/GATK`, `GRCh37/hg19`, `T2T CHM13-v2.0/hs1`, and a custom genome configured via `reference_paths.json` (see Configuration).

When the IGV module is opened from the fusion gene module, the viewer reuses the genome chosen during upload in Step 1, so it matches the reference used for the fusion snapshots. If **Don't create IGV snapshots** was selected during upload, the same genome selector is shown in the module before the viewer loads. —

Bookmarks Table

Above the IGV viewer, a bookmarks table lists all flagged variants or fusions for the selected patients. Clicking any row in the table navigates the IGV viewer directly to the genomic position of that variant or fusion breakpoint. For fusions, both breakpoint positions are shown and the viewer jumps to a split view of both loci simultaneously.

Loading and Reloading

Click **Load IGV Viewer** to initialise the browser with the current tracks and locus. If you change the patient selection after IGV has already been loaded, a dialog will ask whether you want to reload the viewer with the new patient data or continue with the current view.

> IGV.js is a web-based implementation of the IGV desktop application. While it covers the essential functionality needed for read-level validation, some advanced features of the desktop version are not available.

Network Graph

The Network Graph module provides an interactive visualisation of protein-protein interactions within biological pathways, contextualising molecular alterations identified in previous modules. The nodes of the network are the genes belonging to a selected KEGG (https://www.genome.jp/kegg/) pathway. The edges are the protein-protein interactions between those genes, retrieved from STRING DB (https://string-db.org/). The graph is rendered with Cytoscape.js.

The module consists of two interconnected views: a **main graph** showing all genes within a selected pathway, and a **subgraph** showing a focused selection of nodes for detailed analysis.

> **The Network Graph module requires expression data.** If no expression files were provided during upload, the Network Graph module will not be available.

Graph Elements

Nodes represent genes. They are coloured by `log2FC` value from the expression data:

- Red (increasing intensity) — upregulated genes (`log2FC > 1`)
- White — no significant change
- Blue (increasing intensity) — downregulated genes (`log2FC < -1`)

Node borders are coloured to highlight variants and fusions flagged in previous modules:

- Toggle **Add somatic variants**, **Add germline variants**, or **Add fusions** using the switches above the main graph to highlight the corresponding genes with distinctive border colours.

> **Tip:** Use variant highlighting to quickly identify genes with actionable mutations within pathway contexts.

Edges represent STRING DB protein-protein interactions. Two display modes are available:

- **Confidence** (default) — one edge per gene pair, with edge width reflecting the combined confidence score
- **Evidence** — multiple edges per gene pair, one for each type of supporting evidence (experiments, databases, textmining, coexpression, neighborhood, gene fusion, cooccurrence)

Controls

Pathway and Layout

- **Pathway** — select a KEGG pathway to display. Only pathways relevant to the patient's expression data are shown.
- **Layout** — choose between `cola` (recommended for smaller graphs, up to ~100 nodes) and `fcose` (recommended for large complex networks).
- **Tissue** — when multiple reference tissues are available, select which tissue's `log2FC` values are used for node colouring.

Edge Filtering

- **Interaction sources** — select which types of STRING evidence to include in the network. All sources are selected by default.
- **Interaction score** — set a minimum confidence threshold for displayed edges. Available levels: lowest confidence (0.15), medium (0.4, default), high (0.7), highest (0.9). Higher thresholds produce fewer but more reliable interactions.

> **Tip:** Adjusting the confidence threshold significantly changes network density. Start with medium confidence (0.4) and increase if the graph is too dense to interpret.

Graph Manipulation

Three buttons are available below the main graph:

- **Fit graph** — adjusts the view to fit all nodes within the visible area. If nodes are selected, fits the selection instead.
- **Clear selection** — deselects all selected nodes.
- **Select first neighbors** — selects all nodes directly connected to the currently selected node, useful for exploring the local network neighbourhood.

Nodes can be dragged to reposition them. Click a node to select it; click again to deselect.

Subgraph

The subgraph is created automatically when you select one or more nodes in the main graph. It shows the selected nodes and their interactions in an isolated view.

Additional genes can be added to the subgraph by entering gene names in the **Add new genes** field, even if they are not present in the currently selected pathway. Nodes can be removed from the subgraph using the **Remove nodes** field. Both the main graph and subgraph share the same edge display settings and confidence thresholds.

> **Note:** When adding genes not present in the original pathway, ensure that gene names are correctly formatted to avoid unrecognised entries.

Data Tables

Both the main graph and the subgraph have associated tables below them showing gene name, `log2FC`, p-value, pathway name, and number of pathways the gene participates in. Clicking a row in a table highlights the corresponding node in yellow in the graph.

Summary and Report

The Summary and Report module provides a consolidated overview of all findings flagged in previous modules and enables generation of a clinical report in `.docx` format.

Overview Boxes

At the top of the page, four colour-coded info boxes summarise key metrics for each dataset:

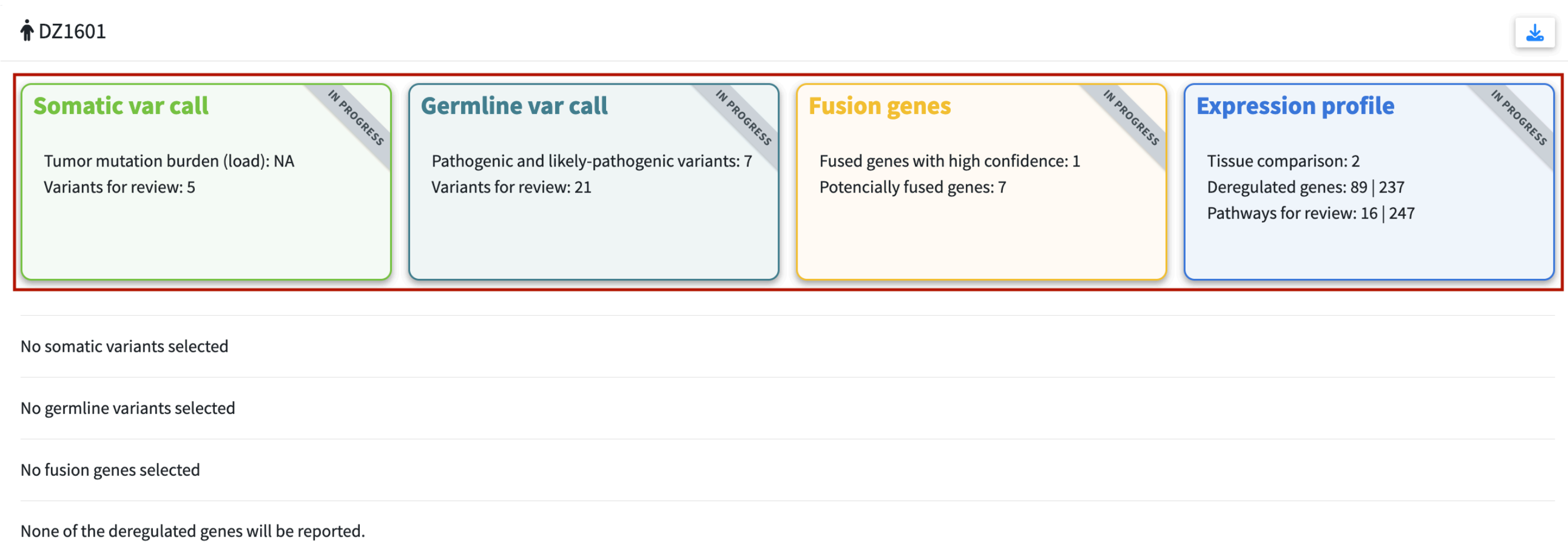

Module	Metrics shown
**Somatic variant calling**	Tumour mutational burden (TMB); number of variants passing default filters
**Germline variant calling**	Number of variants passing default filters; number of pathogenic and likely-pathogenic variants by ClinVar
**Fusion genes**	Number of high-confidence fusions (Arriba); total number of detected fusions
**Expression profile**	Number of reference tissues; number of deregulated genes; number of altered pathways

Each box also carries a small diagonal ribbon in its top corner that mirrors the completion state set in the analysis module. While a dataset is still under review, the ribbon is pale grey and reads in progress. Once the analysis is marked complete, the ribbon switches to the module's own colour and reads complete. This gives a quick sense of how far the case has been worked through before the report is generated.

If a dataset was not loaded, its box is greyed out and shows N/A values.

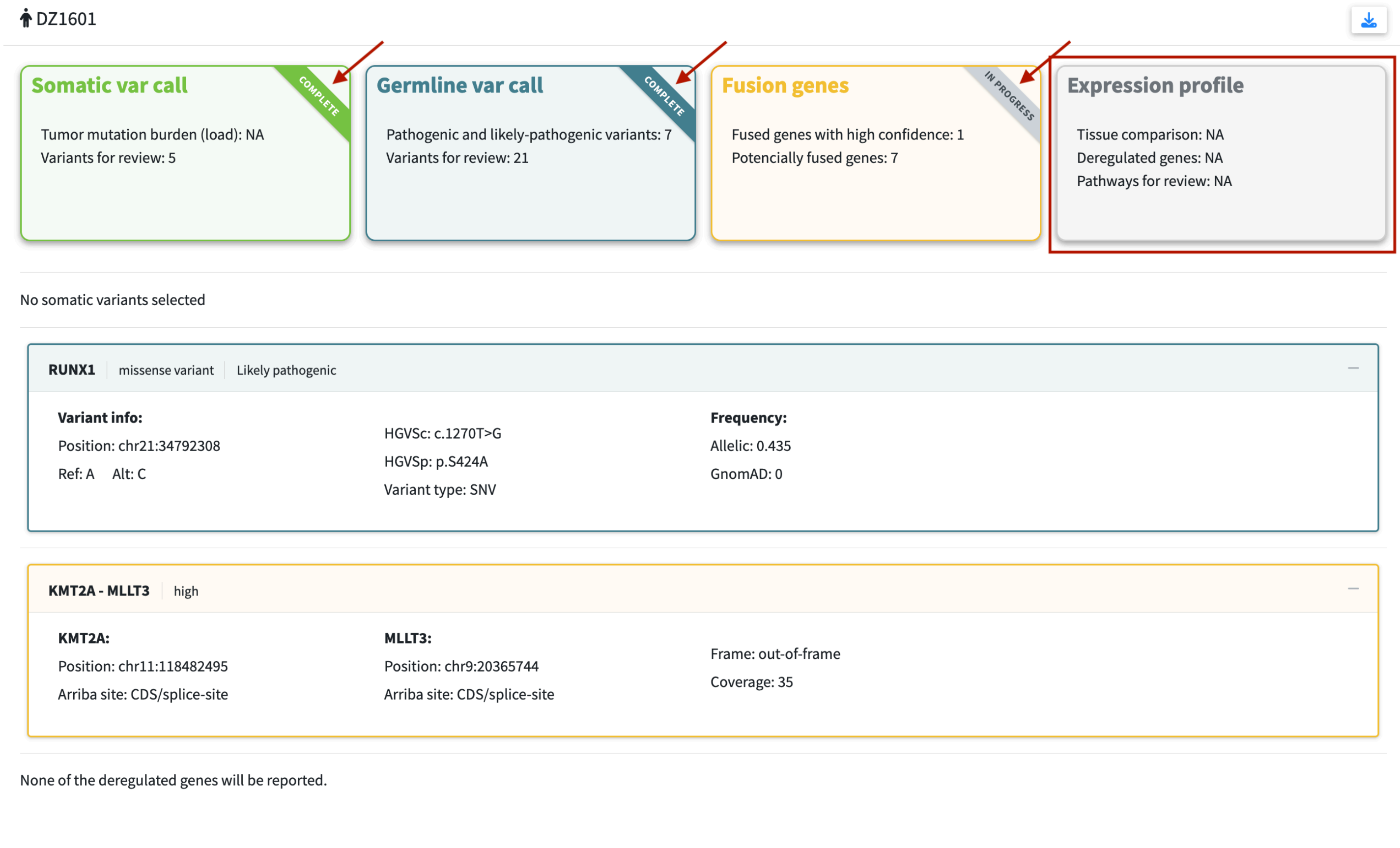

Selected Findings

Below the overview boxes, all flagged variants, fusions, and genes from previous modules are displayed as expandable panels, one per dataset type.

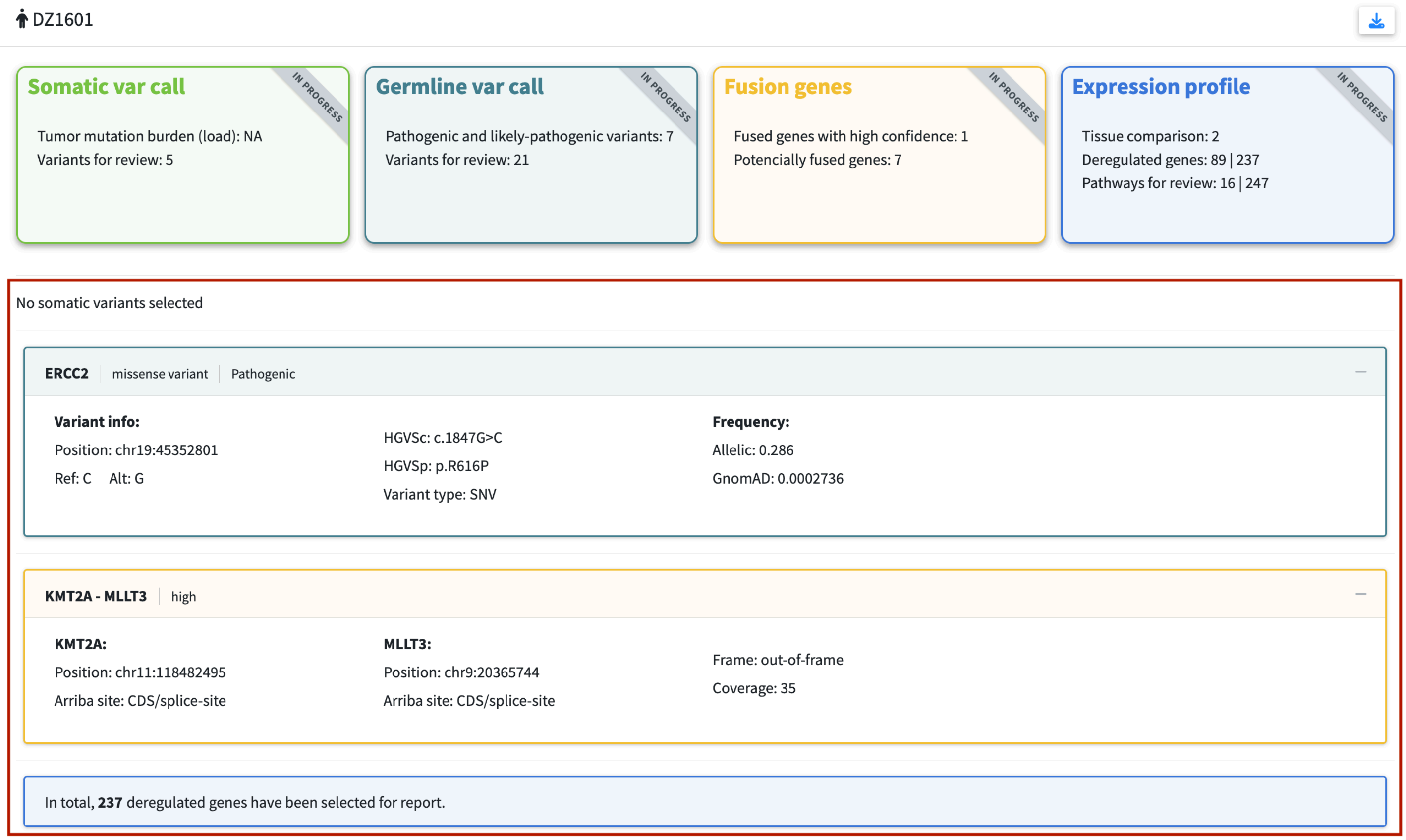

Somatic variants — the panel header shows the gene name and consequence. Expanding reveals:

- *Variant info:* chromosomal position, Ref/Alt alleles, HGVSc, HGVSp, variant type
- *Frequency:* allelic frequency (VAF), gnomAD NFE frequency

Germline variants — the panel header shows gene name, consequence, and ClinVar significance. Expanding reveals:

- *Variant info:* chromosomal position, Ref/Alt alleles, HGVSc, HGVSp, variant type
- *Frequency:* allelic frequency, gnomAD NFE frequency

Fusion genes — the panel header shows the gene pair and Arriba confidence level. Expanding reveals:

- For each fusion partner: position and Arriba site annotation
- Reading frame (Arriba)
- Overall support (read coverage)

Expression profile — not expandable. Shows the total number of deregulated genes selected for the report (combining flagged genes from both the Genes of Interest and All Genes tabs).

Generating the Report

Click the download icon in the top right of the module to open the report options. Two template options are available:

- **Default template** — the built-in template configured via `reference_paths.json` (see Configuration)
- **Custom template** — upload your own `.docx` file with institutional formatting

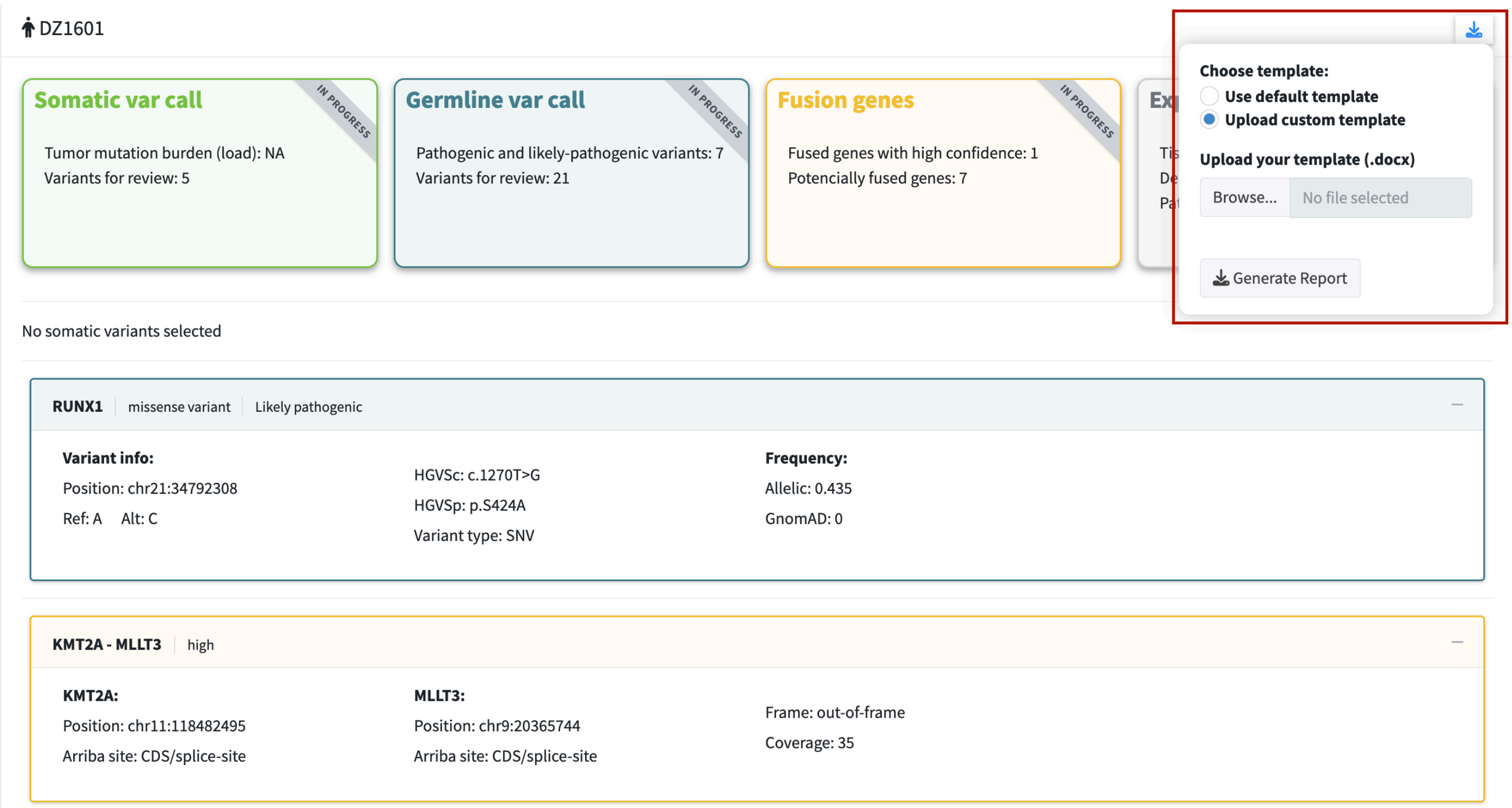

Click **Generate Report** to download the populated `.docx` file.

> The automatically generated report should be regarded as a **draft**. It requires manual review and completion by a clinician before use in a clinical context.

Custom Templates

Custom templates use keyword-based variable substitution. Place keywords in your `.docx` template where you want the corresponding content to appear — the application replaces each keyword with the relevant patient data when generating the report.

All keywords must be wrapped in double angle brackets exactly as shown.

Keyword	Content inserted
<<patient_info>>	Patient ID and report generation date
<<details_table>>	Empty sample details table (specimen type, collection date, method, library preparation, sequencing device, date of sequencing) — one column per loaded dataset, to be filled in manually
<<summary_somatic>>	Short text list of flagged somatic variants in format `Gene (HGVSc/HGVSp)`
<<summary_germline>>	Short text list of flagged germline variants in format `Gene (HGVSc/HGVSp)`
<<summary_fusion>>	Short text list of flagged fusions in format `Gene1::Gene2 gene fusion`
<<somatic_table>>	Detailed somatic variant table: Gene, Transcript, Variant (HGVSc/HGVSp), VAF, Variant effect, Class
<<germline_table>>	Detailed germline variant table: Gene, Transcript, Variant (HGVSc/HGVSp), MAF, Variant effect, Phenotype, Zygosity, Inheritance, Class
<<fusion_table>>	Detailed fusion table: Gene 5', Transcript 5', Gene 3', Transcript 3', Overall support, Phasing
<<expression_table>>	Detailed expression table grouped by pathway: Gene, Transcript, Expression level, log2FC, Therapeutic option
<<mutation_burden>>	Tumour mutational burden value in mutations/Mb
<<somatic_interpretation>>	One sentence per flagged somatic variant with a link to the OncoKB gene page
<<germline_interpretation>>	One sentence per flagged germline variant with a link to the OncoKB gene page

If a keyword is present in the template but no data is available for that section (e.g. no fusions were flagged), the placeholder is replaced with a default message rather than left empty. Keywords not present in the template are simply ignored.

Tip: Test your template with sample data before using it in a clinical workflow to ensure all placeholders are substituted correctly and the formatting is preserved.

Configuration

SeqUIaSCOPE reads a `reference_paths.json` file at startup to locate reference files and configure optional features. A default version of this file is embedded in the Docker image. If you need to override any of the defaults — for example to use a custom report template or a custom reference genome — mount your own `reference_paths.json` into the container.

Mounting a Custom Config

Add the following volume mount to your `docker-compose.yaml`:

```
volumes:
  - ./input_files:/input_files:ro
  - ./output_files:/output_files
  - ./reference_paths.json:/srv/shiny-server/sequiaScope/app/reference_paths.json:ro
```

Place your `reference_paths.json` in the same directory as the compose file.

Config File Structure

```
{
  "output_dir": "output_files",
  "reference_files": {
    "kegg_tab": "app/references/kegg_tab.tsv",
    "report_template": "app/references/report_template.docx",
    "genes_of_interest": "app/references/genes_of_interest.tsv"
  },
  "custom_genome": {
    "display_name": "Custom genome",
    "igv_id": "custom",
    "genome": "custom_genome/GRCh38.fa",
    "index": "custom_genome/GRCh38.fa.fai"
  }
}
```

Configuration Options

`output_dir`

Directory where session data, IGV snapshots, and Arriba reports are stored. Defaults to `output_files`. In Docker deployments this should match the writable volume mount.

`reference_files`

Paths to the three built-in reference files. All paths are relative to the application root inside the container.

Key	Default	Description
`kegg_tab`	`app/references/kegg_tab.tsv`	KEGG pathway annotation table used by the Network Graph module and expression pathway assignment. Required when using expression or network graph features.
`report_template`	`app/references/report_template.docx`	Default `.docx` template used for report generation in the Summary module. Can be replaced with an institutional template.
`genes_of_interest`	`app/references/genes_of_interest.tsv`	Default genes of interest list used for expression highlighting. See Data Requirements — Genes of Interest for the required file format.

`custom_genome`

Optional section for configuring a custom reference genome for IGV. Remove this section entirely if you are using one of the built-in genomes (`GRCh38/hg38`, `GRCh37/hg19`, etc.).

Key	Description
`display_name`	Name shown in the genome picker in the upload form
`igv_id`	Internal identifier — must be set to `"custom"`
`genome`	Path to the FASTA file, relative to the `/input_files` mount
`index`	Path to the FASTA index (`.fai`), relative to the `/input_files` mount

Custom genome files must be placed inside your `input_files` directory so they are accessible via the `/input_files` mount. Paths in the config are relative to that mount point.
